# Deubiquitination-related genes define immune subtypes of colorectal cancer and are associated with prognosis and immunotherapy-related signatures

**DOI:** 10.1038/s41598-026-35271-5

**Published:** 2026-01-08

**Authors:** Yiwei Xu, Zhiyong Mo, Qing Jiang, Jingjing Pan, Qi Xu, Juwen Jia

**Affiliations:** 1 Department of General Surgery, Second People’s Hospital of Longgang District, No. 3 Chuangzuo Road, Buji Street, Longgang District, Shenzhen, 518112 Guangdong China; 2 Department of Oncology, West Coast Second Hospital, Qingdao University Medical Group, Qingdao, Shandong China

**Keywords:** Colorectal cancer, Deubiquitination, Molecular subtypes, Immune microenvironment, Immunotherapy, Prognostic biomarkers, Biomarkers, Cancer, Computational biology and bioinformatics, Immunology, Oncology

## Abstract

Colorectal cancer (CRC) is highly heterogeneous, and the contribution of deubiquitination (DUB) programs to immune context and clinical outcome remains incompletely defined. We integrated transcriptomes from TCGA and GTEx (COAD/READ) with an external GEO cohort (GSE39582) to delineate DUB-associated features. Differential expression and univariate survival analyses yielded DUB-related, prognosis-associated genes, which were used for consensus clustering to derive CRC subtypes. Functional enrichment (GO/KEGG) and preranked GSEA characterized pathways; a STRING–Cytoscape–cytoHubba workflow identified hub genes; immune landscapes were profiled by CIBERSORT together with immune-checkpoint, immunogenic cell death (ICD) and HLA gene sets, and computational immunotherapy-related metrics (TIDE, IPS). Seventeen key DUB-related genes enriched in cell-cycle/DNA-damage response and ECM/EMT programs classified tumors into two robust subtypes with distinct overall survival, clinicopathologic profiles and transcriptional signatures. Sixty-six cluster-related DEGs formed an interaction network that highlighted nine ECM-centric hub genes with consistent prognostic value across TCGA and GEO. Subtypes differed markedly in immune-cell infiltration and in ICG/ICD/HLA expression, with clear separation of TIDE and IPS scores, indicating distinct computational immunotherapy-related signatures. These results define interpretable DUB-anchored immune subtypes and nominate hub genes as candidate biomarkers to support prognosis assessment and facilitate hypothesis-generating immunotherapy-related stratification. Prospective and functional validation is warranted, and this study should be interpreted as an exploratory analysis based on publicly available transcriptomic datasets.

**Supplementary Information:**

The online version contains supplementary material available at 10.1038/s41598-026-35271-5.

## Introduction

CRC, which is a highly prevalent and lethal malignancy worldwide, faces three major clinical challenges: the difficulty of early detection, the inadequacy of current stratification systems, and the persistence of therapeutic resistance^1,2^. Although conventional pathology and staging guide initial treatment, they remain insufficient to capture the intrinsic heterogeneity of tumors, which results in divergent prognoses and therapeutic responses among patients with similar staging; consensus molecular subtypes (CMS) have been identified to exemplify such underlying biological divergence^3^. The dysregulation of the deubiquitination (DUB) axis is mechanistically linked to these clinical challenges: aberrant DUB activity may yield early diagnostic biomarkers through genomic instability, drive the emergence of heterogeneous molecular subtypes via distinct pathway dependencies, and promote therapeutic resistance by stabilizing checkpoint and resistance-related proteins^4,5^. These connections highlight the DUB axis as a critical bridge between CRC clinical problems and molecular pathology, underscoring the need for systematic characterization of DUB-related molecular features in CRC^6^.The ubiquitination-deubiquitination system serves as a central axis for maintaining protein homeostasis and signal transduction, playing pivotal roles in regulating the cell cycle and mediating the DNA damage response (DDR), metabolism, and immune microenvironment shaping^4,6^. Previous studies indicate that DUBs drive CRC growth, invasion, and immune evasion by multiple mechanisms: for example, *USP1* regulates chromosomal stability via DDR, *OTUB1* modulates immune checkpoint (*PD-L1*) activation by suppressing its ubiquitin-mediated degradation, and *USP9X* promotes remodeling of the extracellular matrix (ECM) and epithelial-to-mesenchymal transition (EMT)^5^. Despite ongoing advances in basic and translational research on the DUB axis, which suggest therapeutic potential across multiple tumor types, systematic DUB-based molecular network-driven stratification, immune landscape analysis, and external validation remain insufficient in CRC^5^.

Most existing CRC subtyping approaches rely on single-omics or limited genetic features, struggling to balance mechanistic interpretability with cross-cohort robustness^3,7^. Immune-related metrics like TIDE, IPS, or inferred immune cell populations are widely used, yet few studies deeply couple them with deubiquitination-dominated signaling axes to reveal the molecular basis of immune sensitivity differences^8,9^. Additionally, systematic evaluations are lacking regarding whether stable associations exist between the “hub-like” roles of key DUB-related genes in protein-protein interaction (PPI) networks and clinical outcomes, or whether these associations can be replicated in external populations^10–12^.

To address these gaps, we designed a multi-step study based on publicly available multi-cohort data. We systematically defined key molecular ensembles; conducted consistent clustering; analyzed immune infiltration; and selected hub genes for validation. Together, these objectives support a central, testable hypothesis: deubiquitination-driven cell cycle/DDR dysregulation and ECM/EMT remodeling jointly shape the CRC immune microenvironment, influencing subtype-specific prognosis and computational immunotherapy-related signatures. If validated, this hypothesis may provide a rationale for future hypothesis-driven studies exploring combination strategies (e.g., DDR inhibitors with ICIs or stromal-modulating approaches with immunotherapy)^13–17^.

## Results

### Analytical workflow

**Fig. 1:**
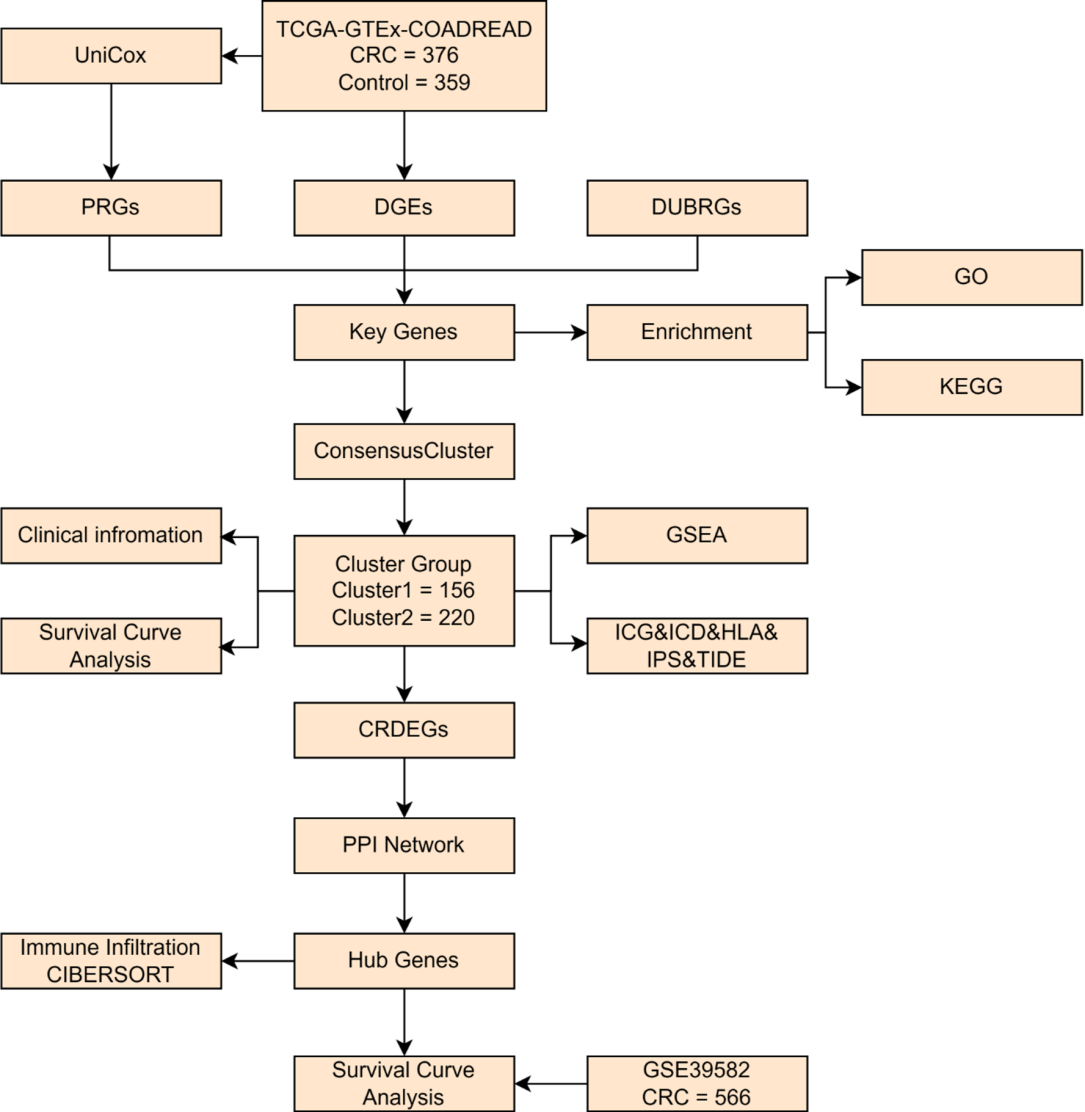
Flow chart for the comprehensive analysis of key genes. *TCGA* the cancer genome atlas, *GTEx* genotype-tissue expression, *COADREAD* colon and rectal adenocarcinoma, *CRC* colorectal cancer, *DEGs* differentially expressed genes, *PRGs* prognosis-related genes, *DUBRGs* deubiquitination-related genes, *CRDEGs* cluster-related differentially expressed genes, *GSEA* gene set enrichment analysis, *GO* gene ontology, *KEGG* Kyoto encyclopedia of genes and genomes, *PPI* protein-protein interaction, *ICG* immune checkpoint genes, *ICD* immunogenic cell death, *HLA* human leukocyte antigen, *IPS* immunophenoscore, *TIDE* tumor immune dysfunction and exclusion.

The overall analytical design is summarized in Fig. 1, which outlines the integrated bioinformatics workflow of this study.

### Prognosis-associated, deubiquitination-related DEGs

**Fig. 2:**
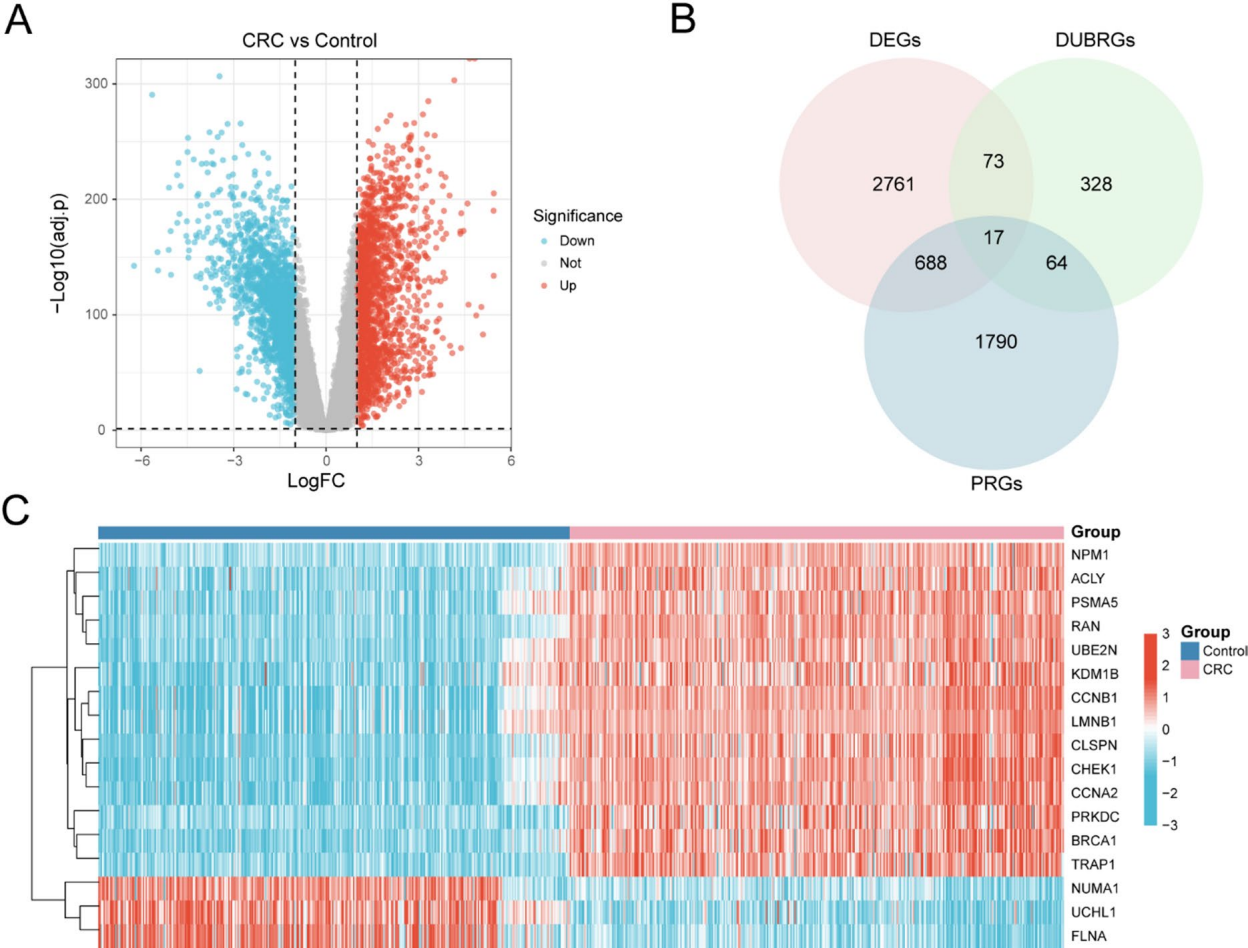
Differential gene expression analysis. Differential expression analysis and identification of key genes in the TCGA-GTEx-COADREAD dataset. (**A**) Volcano plot showing DEGs between CRC and control groups. (**B**) Venn diagram displaying the overlap among DEGs, DUBRGs, and PRGs, yielding 17 key genes. (**C**) Heatmap of key genes in CRC and control samples. Pink indicates CRC group and blue indicates control group; red denotes high expression and blue denotes low expression.

The TCGA-GTEx-COADREAD dataset was divided into CRC tumor and normal control groups. Differential expression analysis between the two groups was performed using the limma package with thresholds of |log_2_FC| > 1 and adjusted *p* < 0.05. In total, 3,539 DEGs were detected, comprising 1763 upregulated (log_2_FC > 1, adjusted *p* < 0.05) and 1776 downregulated (log_2_FC < − 1, adjusted *p* < 0.05) transcripts. A volcano plot summarizing these findings is shown in Fig. 2A.

To obtain the key genes, we performed univariate survival analysis across all genes in TCGA-GTEx-COADREAD and identified 2,559 prognosis-related genes (PRGs) at *p-value* < 0.05 (Supplementary Data S3). We then intersected the DEGs (|log2FC| > 1, adjusted *p <* 0.05), the deubiquitination-related gene set (DUBRGs), and the PRGs, and visualized the overlap with a Venn diagram (Fig. 2B). This yielded 17 key genes: *CHEK1*, *BRCA1*, *CLSPN*, *CCNB1*, *CCNA2*, *TRAP1*, *RAN*, *UCHL1*, *LMNB1*, *PRKDC*, *UBE2N*, *ACLY*, *PSMA5*, *FLNA*, *KDM1B*, *NPM1*, and *NUMA1*. Using this overlap, the expression patterns of the key genes across sample groups in the TCGA-GTEx-COADREAD dataset were examined and displayed as a heatmap produced with the pheatmap package^18^ (Fig. 2C).

### GO and KEGG enrichment analyses

**Fig. 3:**
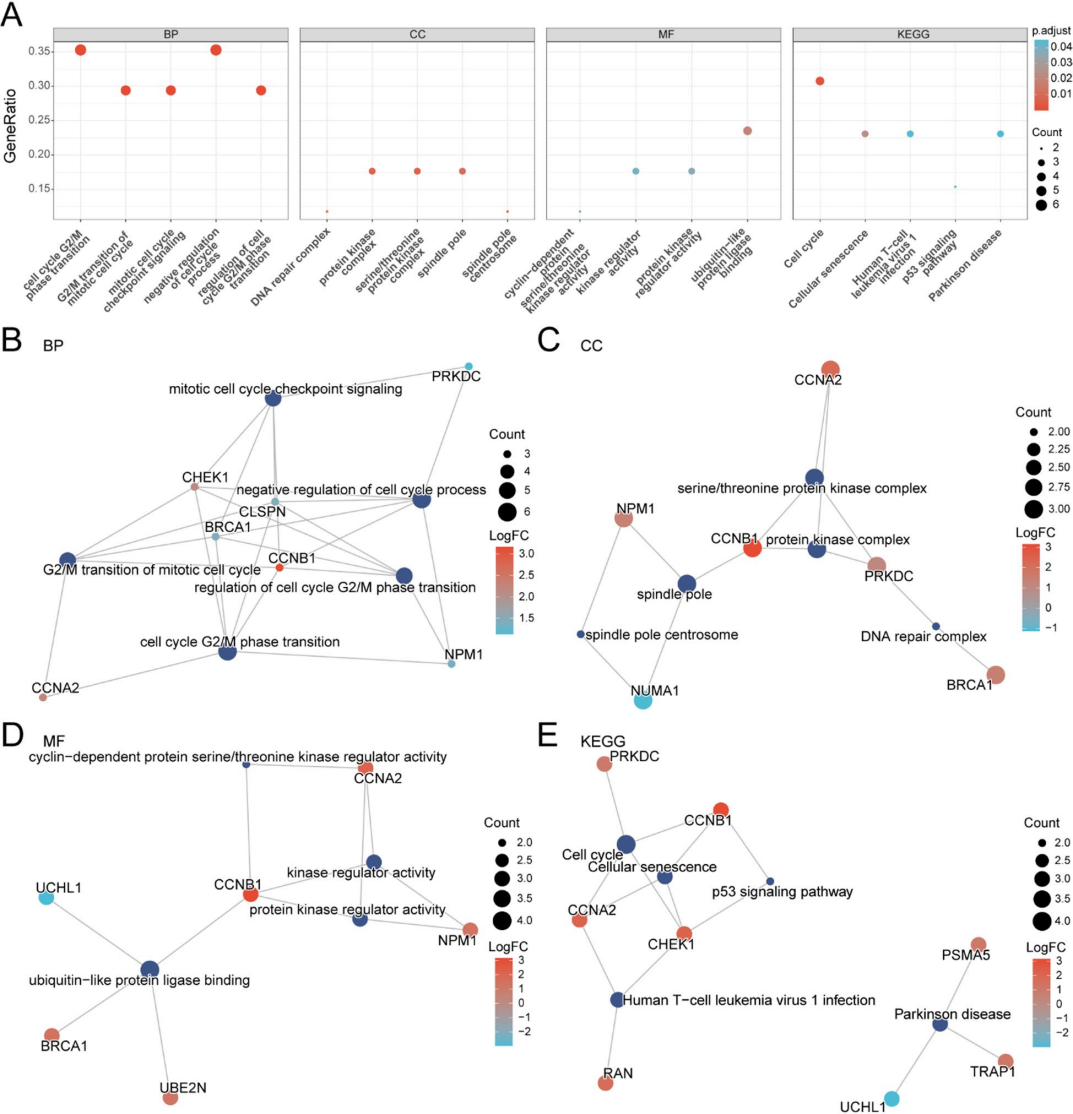
GO and KEGG enrichment analysis of key genes. (**A**) Bubble plot showing enriched GO terms—biological process (BP), cellular component (CC), and molecular function (MF)—and KEGG pathways for key genes. Bubble size indicates the number of genes, and color intensity reflects the adjusted p-value (red = smaller, blue = larger). (**B**–**E**) Network plots of enrichment results: BP (**B**), CC (**C**), MF (**D**), and KEGG pathways (**E**). Node size represents the number of genes involved, and edges indicate shared genes across terms. Data source: KEGG PATHWAY database (https://www.kegg.jp/). KEGG pathway information is cited according to the official KEGG citation policy (https://www.kegg.jp/kegg/kegg1.html).

To elucidate the biological functions of the 17 key genes, GO and KEGG enrichment analyses were performed, covering the categories of BP, CC, and MF, as well as KEGG pathways. Detailed enrichment outcomes are presented in Supplementary Table S2. The 17 genes were mainly enriched in BP terms such as cell cycle G2/M phase transition, regulation of G2/M phase progression, G2/M transition of the mitotic cycle, mitotic checkpoint signaling, and negative regulation of cell cycle processes; CC terms including spindle pole centrosome, DNA repair complex, serine/threonine protein kinase complex, protein kinase complex, and spindle pole; and MF terms such as ubiquitin-like protein ligase binding, cyclin-dependent protein serine/threonine kinase regulator activity, protein kinase regulator activity, and kinase regulator activity. KEGG pathway analysis further revealed significant associations with cellular senescence, cell cycle, human T-cell leukemia virus 1 infection, the p53 signaling pathway, and Parkinson’s disease. The enrichment findings were visualized as bubble plots (Fig. 3A).

In addition, network diagrams were generated for BP, CC, MF, and KEGG results (Fig. 3B-E). Edges indicate annotation links between genes and enriched terms; node size reflects the number of genes associated with a given term. Bubble color encodes the magnitude of log2FC, with more intense red indicating larger positive log2FC values and more intense blue indicating smaller (or negative) log2FC values.

### Construction of CRC subtypes

**Fig. 4:**
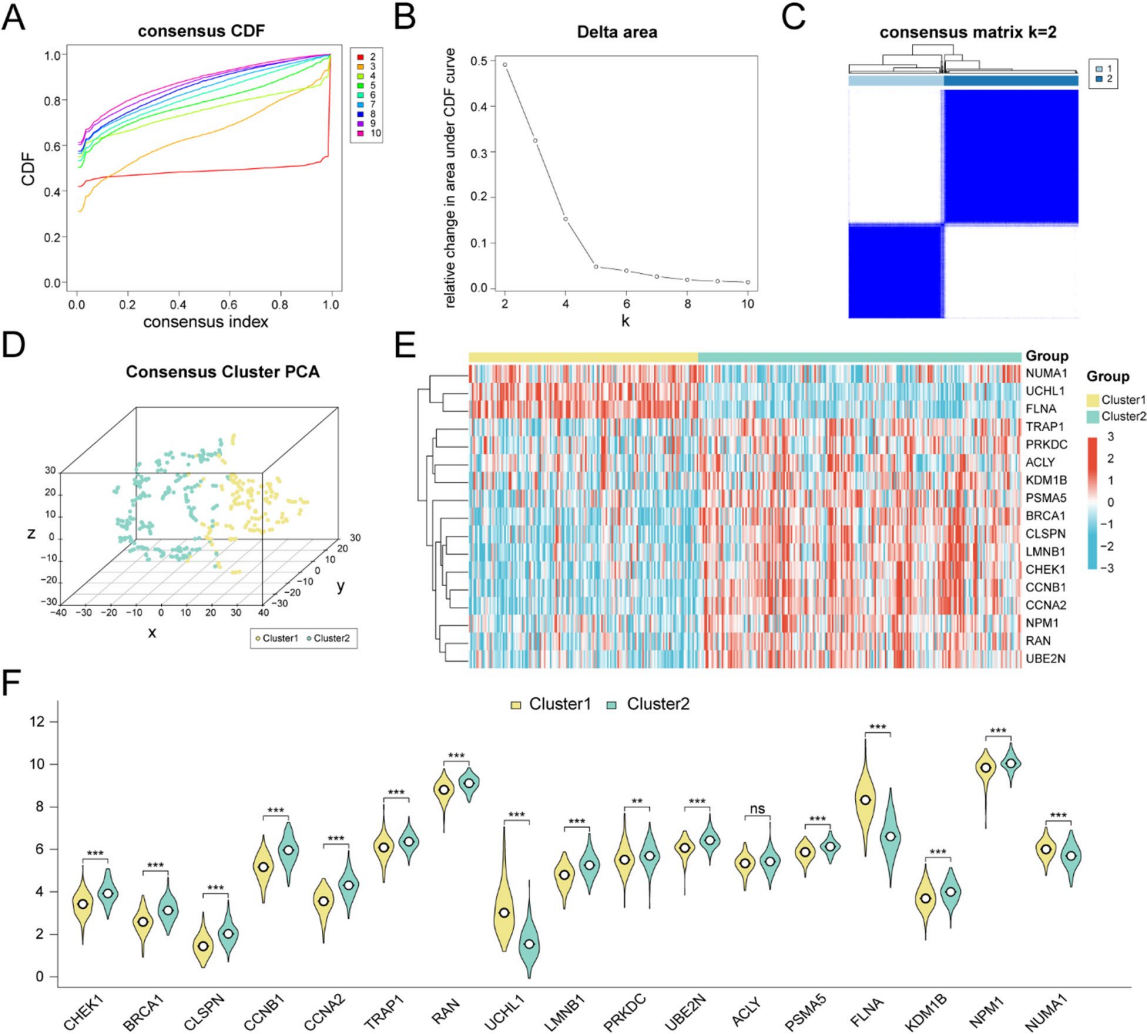
Consensus clustering and subtype characterization in CRC. (**A**,**B**) Cumulative distribution function (CDF) curves and the delta area plot (ΔCDF) used to determine the optimal number of clusters. (**C**) Consensus clustering results for CRC samples in the TCGA-GTEx-COADREAD dataset (k = 2). (**D**) 3D t-distributed stochastic neighbor embedding (t-SNE) visualization of the two disease subtypes. (**E**) Heatmap of expression levels of the 17 key genes across CRC subtypes (red = upregulated, blue = downregulated). (**F**) Group-wise comparisons of the 17 key genes between subtype A (Cluster1, yellow) and subtype B (Cluster2, green); significance annotations: ns, *p* ≥ 0.05; **, *p* < 0.01; ***, *p* < 0.001.

To stratify disease subtypes in the TCGA-GTEx-COADREAD cohort, consensus clustering was carried out with the ConsensusClusterPlus package using the expression patterns of the 17 key genes. Two stable CRC subtypes were defined (Fig. 4A-C): subtype A (Cluster1; *n* = 156) and subtype B (Cluster2; *n* = 220). Three-dimensional t-distributed stochastic neighbor embedding (t-SNE) analysis further confirmed a clear separation between the two subtypes (Fig. 4D).

We next visualized expression differences of the 17 key genes across subtypes using heatmaps generated with pheatmap (Fig. 4E). Finally, group comparison plots (Fig. 4F) showed that 15 key genes exhibited highly significant differences between subtype A (Cluster1) and subtype B (Cluster2) (*p <* 0.001), namely *CHEK1*, *BRCA1*, *CLSPN*, *CCNB1*, *CCNA2*, *TRAP1*, *RAN*, *UCHL1*, *LMNB1*, *UBE2N*, *PSMA5*, *FLNA*, *KDM1B*, *NPM1*, and *NUMA1*. *PRKDC* also differed significantly between the two subtypes (*p <* 0.01).

### Survival curves and clinical characteristics of CRC subtypes

**Fig. 5:**
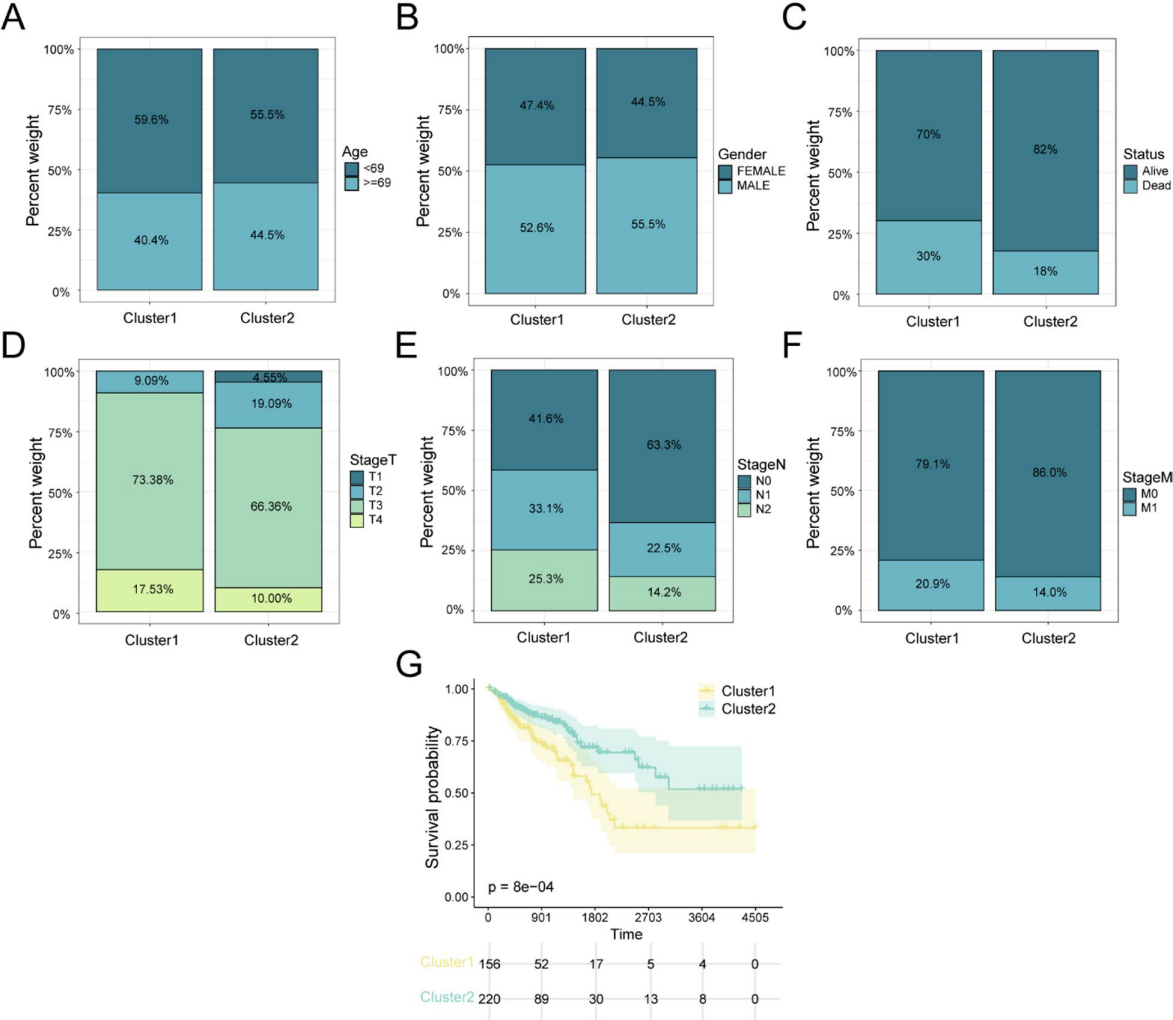
Baseline clinical characteristics and survival analysis of CRC subtypes. (**A**–**F**) Distribution of clinical variables between subtype A (Cluster1) and subtype B (Cluster2): age (**A**), gender (**B**), survival status (**C**), T stage (**D**), N stage (**E**), and M stage (**F**). (**G**) KM survival curves comparing OS between subtype A (Cluster1, yellow) and subtype B (Cluster2, green) in the TCGA-GTEx-COADREAD dataset.

We visualized the distributions of age (Age), sex (Gender), survival status (Status), and clinical staging (TStage, NStage, MStage) across subtypes using stacked bar charts (Fig. 5A-F). Compared with subtype A (Cluster1), subtype B (Cluster2) showed higher proportions of patients aged ≥ 69 years, male sex, alive status, T1-T2 tumors, N0 tumors, and M0 tumors. KM survival analysis comparing subtype A (Cluster1) and subtype B (Cluster2) in the TCGA-GTEx-COADREAD cohort revealed a marked difference in OS (*p* < 0.001) (Fig. 5G).

### Identifying gene expression and pathway alterations across CRC subtypes

To compare the transcriptional profiles of subtype A (Cluster1) and subtype B (Cluster2) in the TCGA-GTEx-COADREAD cohort, differential expression analysis was performed using the limma package. With thresholds set at |log_2_FC| > 1.5 and adjusted *p* < 0.05, 66 CRDEGs were identified, comprising 64 upregulated (log_2_FC > 1.5, adjusted *p* < 0.05) and 2 downregulated (log_2_FC < − 1.5, adjusted *p* < 0.05) transcripts (Supplementary Data S4). A volcano plot summarizing these findings is presented in Supplementary Fig. S1A. Expression differences of the CRDEGs between the two subtypes were further visualized as a heatmap generated with the pheatmap package (Supplementary Fig. S1B).

To assess how the global gene-expression landscape relates to biological programs in CRC, we conducted a preranked GSEA on all genes in the TCGA-GTEx-COADREAD cohort and displayed the results with ridge plots (Supplementary Fig. S1C); detailed outputs are provided in Supplementary Table S3. Notably, the enriched signatures included Foroutan Prodrank Tgfb Emt Up (Supplementary Fig. S1D), Mebarki Hcc Progenitor Wnt Up (Supplementary Fig. S1E), Jechlinger Epithelial to Mesenchymal Transition Up (Supplementary Fig. S1F), and Winter Hypoxia Dn (Supplementary Fig. S1G), along with other biologically meaningful programs and pathways.

### PPI network construction and hub gene identification

The STRING database was queried to build a PPI network for the 66 CRDEGs (Supplementary Fig. S2A). The resulting network included 30 CRDEGs with at least one interacting partner. These 30 genes were subsequently ranked using the cytoHubba plugin in Cytoscape through five topological algorithms—MCC, Degree, MNC, EPC, and Closeness. For each algorithm, the top 10 ranked genes were visualized as PPI subnetworks (MCC, Supplementary Fig. S2B; MNC, Supplementary Fig. S2C; Degree, Supplementary Fig. S2D; EPC, Supplementary Fig. S2E; Closeness, Supplementary Fig. S2F), with node color transitioning from red to yellow to represent decreasing scores. Finally, the overlap among the five top-10 gene sets was summarized in a Venn diagram (Supplementary Fig. S2G), resulting in the identification of nine hub genes for CRC: *COL1A1*,* FN1*,* COL1A2*,* LUM*,* MYH11*,* DCN*,* COL3A1*,* POSTN*, and *BGN*.

### Survival analysis and validation of hub genes

**Fig. 6:**
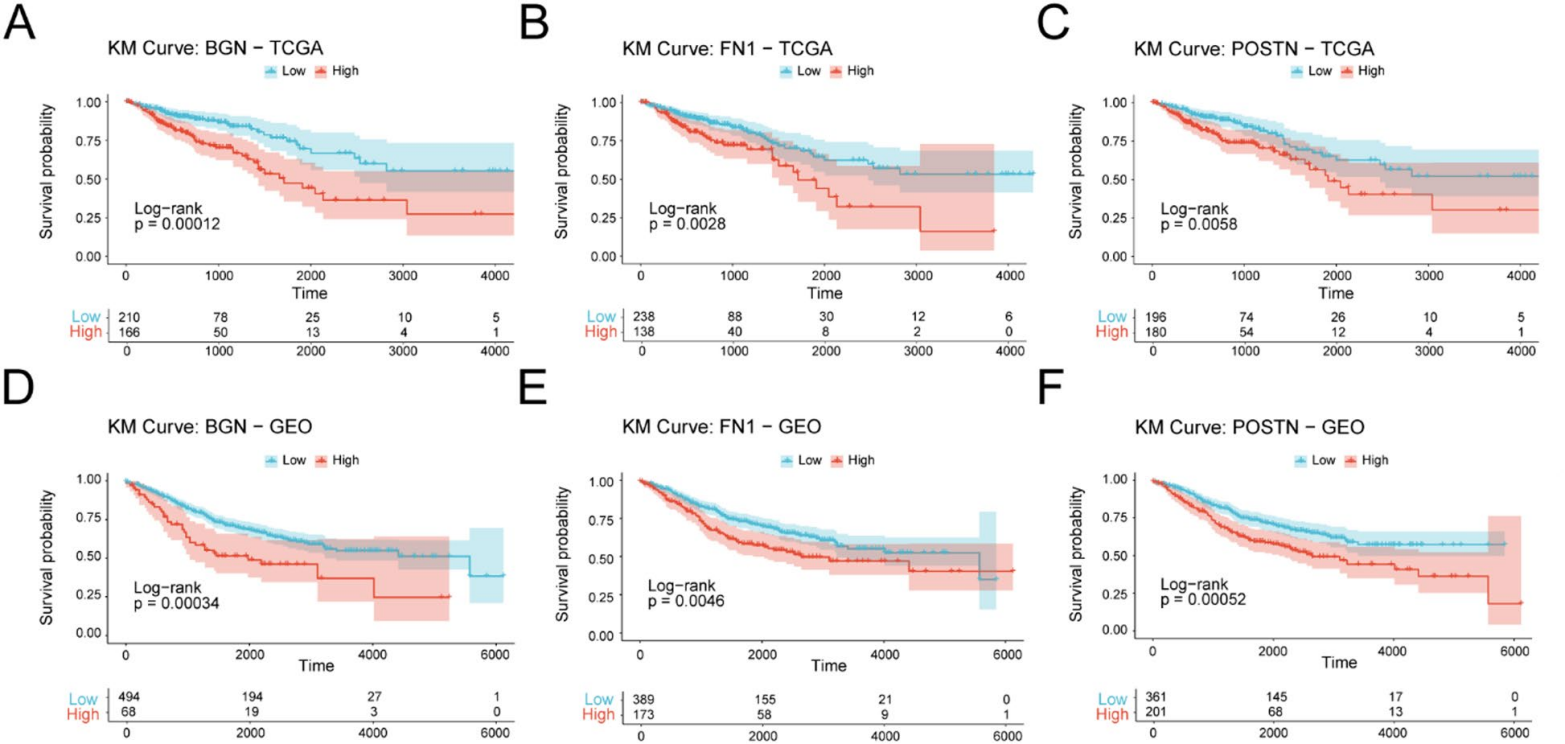
Representative survival validation of ECM-related hub genes across two cohorts. (**A**–**C**) TCGA-COADREAD cohort: Kaplan–Meier curves comparing overall survival between high- and low-expression groups for BGN (**A**), FN1 (**B**), and POSTN (**C**). (**D**–**F**) GSE39582 cohort: independent validation of the same genes (BGN, FN1, POSTN) with concordant risk separation. Red indicates the high-expression group and blue indicates the low-expression group. Survival differences were evaluated using the log-rank test. Full KM results for all nine hub genes (COL1A1, COL1A2, LUM, MYH11, COL3A1, DCN, POSTN, FN1, BGN) are provided in Supplementary Fig. S3-4.

We performed Kaplan–Meier (KM) survival analyses by stratifying tumors into high- versus low-expression groups for each hub gene using a minimum-p-value–based dichotomization. Representative results are shown in (Fig. 6A–F), and full results for all nine hub genes are provided in Supplementary Fig. S3-4. In the TCGA-COADREAD cohort, higher expression of BGN, FN1, and POSTN was associated with significantly worse overall survival (OS; log-rank *p* < 0.01; Fig. 6A–C). The GSE39582 cohort demonstrated consistent prognostic separation for these genes (log-rank *p* < 0.01; Fig. 6D–F). Additional hub genes (*COL1A1*, *COL1A2*, *LUM*, *MYH11*, *COL3A1*, and *DCN*) also showed significant associations with OS in one or both cohorts (Supplementary Fig. S3-4).

### Immune landscape and computational immunotherapy-related metrics across DUB-associated CRC subtypes

**Fig. 7:**
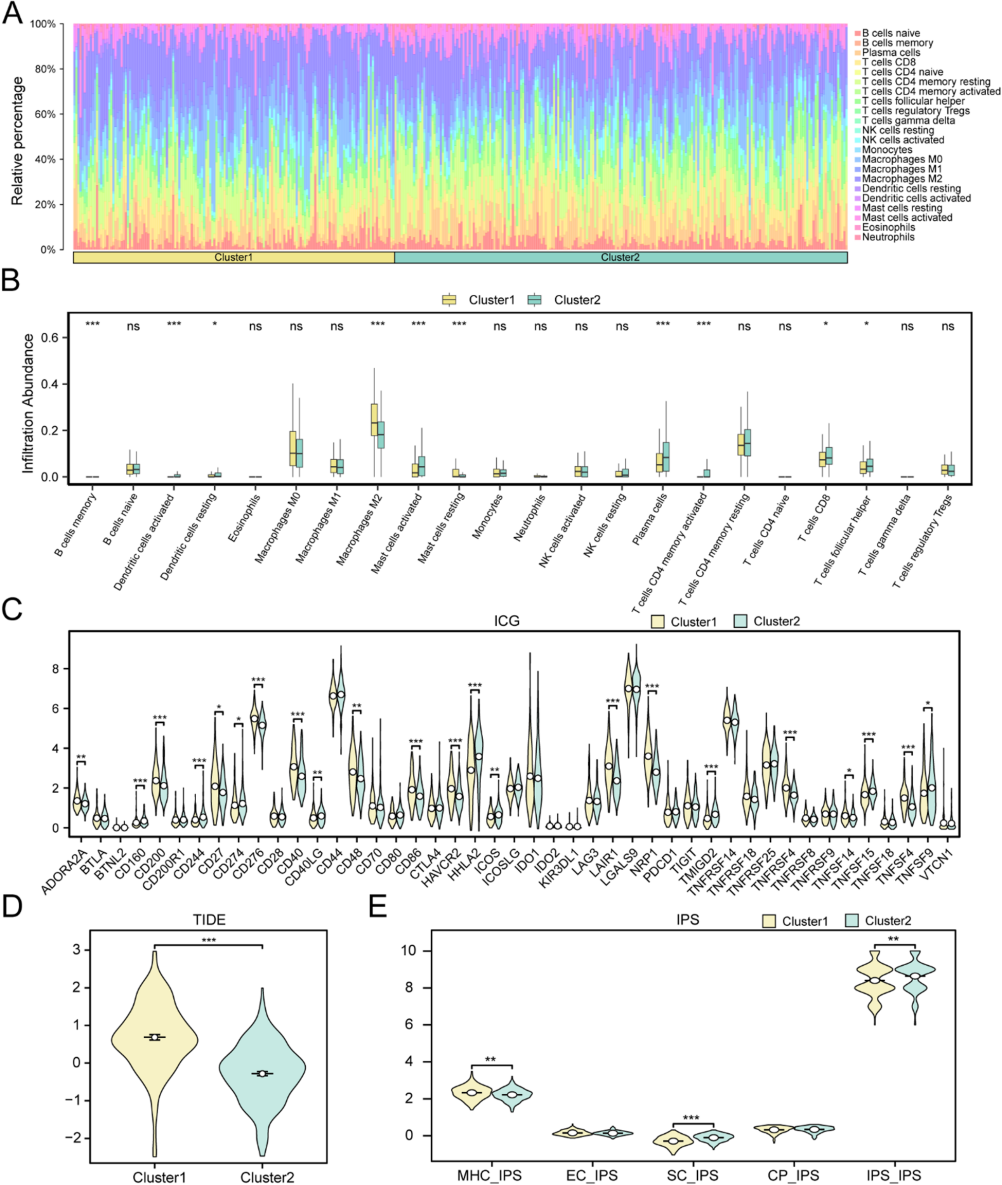
Immune landscape between CRC molecular subtypes. (**A**) Stacked bar plot showing the overall composition of 22 immune-cell populations estimated by CIBERSORT across CRC samples, grouped by subtype. (**B**) Between-subtype comparisons of representative immune-cell fractions; boxes depict the distribution within each subtype. (**C**) Differential expression of immune checkpoint genes (ICGs) between subtypes. (**D**) Comparison of TIDE scores between subtypes. (**E**) Comparisons of IPS-derived immunogenicity scores (MHC, EC, SC, CP and overall IPS) between subtypes. Unless otherwise specified, between-group differences were evaluated using the Wilcoxon rank-sum test. Significance: ns, *p* ≥ 0.05; *, *p* < 0.05; **, *p* < 0.01; ***, *p* < 0.001. Color scheme: yellow = subtype A (Cluster 1), green = subtype B (Cluster 2). Extended results (ICD/HLA gene sets, immune-cell correlation heatmaps and gene–immune associations) are provided in the Supplementary Information.

Using the TCGA–GTEx–COADREAD expression matrix, we applied CIBERSORT to estimate the relative abundances of 22 immune-cell populations. A stacked bar plot summarized subtype-specific immune compositions (Fig. 7A), and between-group comparisons revealed distinct infiltration profiles between subtype A (Cluster 1) and subtype B (Cluster 2), with representative differences in CD8⁺ T cells, T follicular helper (Tfh) cells, and M2 macrophages (Fig. 7B). Immune checkpoint genes (ICGs) also differed significantly between subtypes, indicating divergent immune-activation/immune-suppression states (Fig. 7C). To approximate immunotherapy responsiveness, TIDE scores showed clear separation between subtypes (Fig. 7D), and IPS-derived immunogenicity scores (MHC, EC, SC, CP, and overall IPS) were likewise distinct (Fig. 7E). Extended visualizations (Supplementary Fig. S5)—including immune-cell correlation heatmaps, gene–immune association plots, and detailed ICD/HLA results—are provided in the Supplementary Information.

## Discussion

This study explores the heterogeneity of deubiquitination (DUB)-related transcriptional programs in CRC. Through a multi-step analysis, two robust molecular subtypes were identified, exhibiting systematic differentiation in OS, immune microenvironment, and immunotherapy-related transcriptomic signatures. Collectively, a “stromal activation-immune rejection” axis centered on ECM/EMT and matrix remodeling interacts with a “proliferation–stress repair” axis associated with DUB-related regulation and involving cell cycle/DDR, shaping distinct prognostic and immune-related transcriptomic landscapes^5,6^. Given the pivotal role of deubiquitination in regulating protein stability, DDR, and immune signaling, elucidating its contribution to CRC subtypes is essential for understanding tumor progression and treatment-related heterogeneity at the transcriptomic level.

It should be noted that among the 17 key genes identified in this study, only a limited subset, such as UCHL1, possesses well-characterized deubiquitinase enzymatic activity. The majority of the remaining genes are more appropriately considered as substrates, regulators, or functional partners within deubiquitination-related signaling pathways rather than catalytic DUB enzymes per se. For example, CHEK1, BRCA1, and PRKDC are core components of cell cycle regulation and the DNA damage response, where they interact functionally with multiple deubiquitinating enzymes to maintain protein stability and checkpoint control. Similarly, genes such as UBE2N and NPM1 participate in ubiquitin chain assembly or protein homeostasis, thereby indirectly modulating deubiquitination-dependent signaling. Together, these observations suggest that the DUB-related transcriptional programs defined here reflect not only alterations in deubiquitinating enzymes themselves but also broader perturbations of downstream signaling networks that are closely linked to tumor progression and immune microenvironment regulation in colorectal cancer.

The poor prognosis subtype exhibits significant enrichment of stromal and fibrotic signaling, including synergistic enhancement of TGF-β-driven EMT upregulation, collagen assembly, hypoxia response, and senescence-related pathways. This aligns with the immune exclusion ecosystem of “matrix densification—physical barrier to immune cells—suppression of effector T cell function”^19,20^. In contrast, the other subtype exhibits weaker matrix remodeling and a more favorable immune-mediated clearance environment, correlating with its superior survival.

Our DUB-associated classification shows comparable mapping to CRC CMS lineages: the subtype enriched in EMT/matrix pathways with poorer prognosis aligns with CMS4 “stromal/mesenchymal” characteristics; while the other subtype aligns more closely with lineages exhibiting balanced immune and epithelial signaling^3,7^. Immunophenotypic decomposition reveals distinct patterns of CD8⁺ T cell, Tfh, and M2 macrophage abundance and co-expression between subtypes, suggesting distinct, subtype-specific immune phenotypic differences^21^; inferences were enhanced using an expression deconvolution framework for robustness^16^.

The two subtypes exhibit systematic differences in HLA family expression, ICD markers, and immune checkpoint (ICG) profiles, along with distinct computational immunotherapy-related signatures reflected by TIDE and IPS scores^8,9^. This aligns with prior evidence that ICD-DAMPs enhance antigen-specific immunity and that computational frameworks are widely used to characterize immunotherapy-related immune features^22^.

These immune-related features and potential therapeutic implications represent transcriptome-based statistical associations and computational inferences and should be interpreted as hypothesis-generating; their biological mechanisms and clinical utility require further experimental validation and independent clinical cohorts.

Multi-algorithm fusion of PPI networks identified a hub gene cluster centered on the matrisome (e.g., *COL1A1/COL1A2/COL3A1*, *FN1*, *POSTN*, *DCN*, *LUM*, *BGN*). These hub genes, by integrating structural remodeling, signal transduction, and immune regulation, contribute to reshaping the mechanical microenvironment and modulating immune cell recruitment and effector activity through the integrin/focal adhesion kinase (FAK)/phosphoinositide 3-kinase (PI3K)/protein kinase B (Akt) signaling axis^23–26^. External validation demonstrated that multiple genes within this network consistently stratified survival in independent GEO cohorts, supporting their potential relevance for prognostic risk stratification.

Previous studies have suggested that several hub genes identified in this study may be indirectly modulated through druggable signaling pathways rather than serving as direct pharmacological targets themselves. For example, FN1 and COL1A1 are key components of the extracellular matrix and can be regulated via the integrin–focal adhesion kinase (FAK)–PI3K/Akt signaling axis. In this context, FAK inhibitors such as defactinib have been reported to attenuate matrix remodeling and stromal activation, thereby reshaping the tumor immune microenvironment. Similarly, POSTN is closely associated with TGF-β signaling, and inhibition of TGF-β receptors (e.g., galunisertib) has been shown to suppress periostin expression and partially alleviate immune exclusion phenotypes. Proteoglycans such as BGN and LUM, as matrix-associated regulators, have also been implicated in extracellular matrix remodeling and immune modulation, suggesting potential susceptibility to pathway-level therapeutic interventions. Together, these findings indicate that hub genes within the DUB-related subtype may represent indirect nodes of pharmacological modulation, providing a conceptual basis for future translational studies targeting stromal–immune interactions.

In alignment with this, combinations of DDR inhibitors and immune checkpoint inhibitors have been explored in preclinical and early clinical settings^27,28^, suggesting a possible direction for future hypothesis-driven research in DUB-proliferation–active subpopulations, pending further experimental and clinical validation.

These statements are intended to outline research directions rather than to make clinical treatment recommendations.

Methodologically, this study employs a multi-layered “function-network-immunity” evidence framework to link DUB-associated molecular profiles with the ECM/EMT-immune axis, providing an interpretable classification coordinate system centered on DUBs beyond existing CMS/immune-subtype frameworks. Practically, the identified features can be utilized in two directions. First, for prognosis and follow-up stratification. Second, to provide a conceptual framework for immunotherapy-related molecular stratification and to facilitate hypothesis-driven exploration of potential combination strategies—for example, at the hypothesis-generating level, considering TGF-β/CAF/ECM-targeting combined with PD-1/PD-L1 in “matrix-immune exclusion” patients^19,20,29^, while exploring sequential or concurrent combinations of DDR inhibitors (e.g., PARP) with ICIs in “DDR-proliferation active” patients^27,29^. As a data-science foundation, the integrated molecular characteristics from TCGA-COAD/READ provide a macro-level reference for these observations^30^.

It should also be noted that the present study primarily focused on transcriptome-level analyses to characterize deubiquitination (DUB)-related molecular patterns and their associations with the immune microenvironment in colorectal cancer. Established genomic biomarkers such as tumor mutational burden (TMB) and microsatellite instability (MSI), which are known to influence immunotherapy responsiveness, were not incorporated into the current analytical framework. While these mutation-based features are clinically relevant, they represent a distinct molecular layer beyond the scope of the present study. Our objective was to explore DUB-centered transcriptional programs and their relationship with immune infiltration and immunotherapy-related signatures, rather than to provide a comprehensive multi-omics evaluation of genomic instability.

This study has several limitations that should be acknowledged. Firstly, it is entirely a retrospective computational analysis without experimental confirmation; laboratory assays such as qPCR, Western blotting, or immunohistochemistry are required to validate the expression patterns and mechanistic roles of the identified hub genes. Secondly, although we integrated multiple large cohorts (TCGA, GTEx, and GSE39582), the sample size remains relatively modest, predominantly Western populations, which may limit generalizability to other ethnic groups. Thirdly, clinical validation is lacking, as the predicted immunotherapy responses (TIDE, IPS) have not been tested in prospective CRC cohorts receiving ICIs. Fourthly, despite cross-platform correction, residual batch effects from integrating heterogeneous datasets may still bias the clustering and survival associations. Finally, the absence of methylation, proteomic, metabolomic, and single-cell or spatial transcriptomic data restricts mechanistic depth, and future spatiotemporal multi-omics could better delineate the dynamic processes of matrix remodeling, T-cell mobilization, and antigen presentation.

Based on integrative analyses of transcriptomic data from TCGA, GTEx, and GEO cohorts, this study systematically characterized deubiquitination (DUB)-related molecular patterns and their associations with the immune microenvironment in colorectal cancer. Through consensus clustering, external validation, and multidimensional immune evaluation, we identified robust DUB-associated subtypes exhibiting distinct survival outcomes, immune infiltration profiles, and immunotherapy-related transcriptomic signatures. As a computational exploratory study leveraging large-scale public datasets, this work is intended to generate biologically interpretable hypotheses and stratification frameworks rather than to establish direct causal mechanisms.

Notably, by focusing on the deubiquitination regulatory axis, our study integrates cell cycle/DNA damage response signaling with ECM–EMT-related stromal programs into a unified analytical framework, highlighting their coordinated roles in shaping immune heterogeneity within the tumor microenvironment. This integrative perspective extends beyond existing CMS or immune subtype classifications by providing a DUB-centered, system-level view of tumor–stroma–immune interactions. Collectively, our findings offer a coherent and reproducible stratification model that may facilitate the design of future functional experiments and clinical validation studies aimed at refining prognostic assessment and exploring rational, hypothesis-driven combination therapeutic strategies in colorectal cancer.

## Methods

### Data acquisition and preprocessing

Data for the TCGA-COADREAD cohort were downloaded using the R package TCGAbiolinks^31^ from the Genomic Data Commons (GDC) repository (https://portal.gdc.cancer.gov/*)*, which was used as the training set for subsequent analyses. After excluding cases lacking survival information, we retained 376 CRC tumor samples with survival data and 51 normal control samples with RNA-seq read counts. The data were then normalized as FPKM values, and the associated clinical metadata were retrieved from the UCSC Xena repository^32^ (https://xena.ucsc.edu/*)*, as summarized in Table 1. In addition, 308 normal colorectal tissue samples (RNA-seq read counts) were obtained from the Genotype-Tissue Expression (GTEx) project^13^ via UCSC Xena and normalized to FPKM. These GTEx normals were merged with the TCGA cohort (376 CRC tumors and 51 normals) to construct a combined dataset comprising 376 CRC tumors and 359 normal controls, hereafter referred to as TCGA-GTEx-COADREAD.

**Table 1:** TCGA clinical information.

Characteristics	CRC
n	376
Gender, n (%)	
Male	204 (54.3%)
Female	172 (45.7%)
Age, n (%)	
≥ 69	161 (42.8%)
< 69	215 (57.2%)
OS, n (%)	
0	290 (77.1%)
1	86 (22.9%)
M stage, n (%)	
M0	255 (83.1%)
M1	52 (16.9%)
N stage, n (%)	
N0	202 (54.3%)
N1	100 (26.9%)
N2	70 (18.8%)
T stage, n (%)	
T1	10 (2.7%)
T2	56 (15%)
T3	259 (69.3%)
T4	49 (13.1%)

*TCGA* the cancer genome atlas, *CRC* colorectal cancer.

All analyses in this study were conducted in accordance with relevant guidelines and regulations.

The CRC microarray dataset (GSE39582) was obtained from the Gene Expression Omnibus (GEO) repository^33^ [https://www.ncbi.nlm.nih.gov/geo/] through the R package GEOquery^34^ (v2.70.0). GSE39582 comprises samples from Homo sapiens; the tissue source was Colorectal cancer(CRC) tumor tissue; and the microarray platform was GPL570, with detailed characteristics summarized in Supplementary Table S1. GSE39582 comprised 566 CRC tumor and 19 normal control samples, and all tumor samples were analyzed in this study.

We queried the GeneCards database^35^ (https://www.genecards.org/) to compile a catalog of deubiquitination-related genes (DUBRGs). Using “deubiquitination” as the search term and retaining only protein-coding entries with a relevance score > 1, we obtained 587 DUBRGs.

In this study, the term “deubiquitination-related genes” refers to a curated gene set associated with deubiquitination processes and ubiquitin-mediated protein homeostasis. This set includes not only canonical deubiquitinases with catalytic activity but also genes functionally associated with deubiquitination-related pathways and ubiquitin-mediated protein homeostasis, aiming to capture the broader regulatory network of deubiquitination at the transcriptome level.

In parallel, we searched PubMed (https://pubmed.ncbi.nlm.nih.gov/) using the same keyword and extracted DUBRGs reported in published studies^4–6^, yielding five additional genes. After merging the two sources and removing duplicates, a total of 591 DUBRGs were retained (Supplementary Data S1). Finally, intersecting these genes with those having expression data in the TCGA–GTEx–COADREAD RNA-seq dataset and the GEO dataset(s) resulted in 482 DUBRGs for downstream analyses (Supplementary Data S2).

Batch effects in GSE39582 were adjusted using the R package sva^36^ (v3.50.0). The merged GEO datasets (referred to as Combined Datasets) underwent preprocessing with limma^37^ (v3.58.1), including probe annotation and data normalization.

### Identification of deubiquitination-related and prognosis-associated differentially expressed genes (DEGs) in CRC

Based on the grouping of the TCGA-GTEx-COADREAD dataset, samples were stratified into CRC tumors and normal controls. Differential expression analysis between tumor and control groups was carried out using the *limma* package (v3.58.1)^37^. DEGs were defined as those showing an absolute log_2_ fold change (|log_2_FC|) greater than 1 with a false discovery rate (FDR)-adjusted *p-value* below 0.05. Genes with log_2_FC > 1 and FDR-adjusted *p* < 0.05 were considered upregulated, whereas those with log_2_FC < − 1 and FDR-adjusted *p* < 0.05 were classified as downregulated. Multiple testing correction was conducted using the Benjamini-Hochberg (BH) procedure to control the FDR^38^. Volcano plots were subsequently generated with the ggplot2 package (v3.5.2).

Univariate survival analysis was conducted for all genes in TCGA-GTEx-COADREAD tumor samples employing the R package survival (v3.8.3)^39^. Genes showing p-values below 0.05 were defined as prognosis-associated (PRGs). Key CRC-related genes were determined by overlapping DEGs (|log2FC| > 1, adjusted *p* < 0.05) from TCGA-GTEx-COADREAD with the deubiquitination-related gene set (DUBRGs) and the PRGs, and the intersections were visualized using a Venn diagram. Heatmaps were generated with the pheatmap package (v1.0.13).

### Gene ontology (GO) and Kyoto encyclopedia of genes and genomes (KEGG) functional enrichment analysis

GO^40^ analysis is a widely used framework for comprehensive functional annotation, covering the categories Biological Process (BP), Cellular Component (CC), and Molecular Function (MF). The KEGG^41^ offers a curated knowledge base that integrates genomic, signaling, disease, and pharmacological pathway information. In this study, functional enrichment of the key genes was conducted using the clusterProfiler R package^42^ (v4.10.1) to systematically examine GO and KEGG pathways. Terms were considered statistically significant when the adjusted *p-value* was below 0.05 and the false discovery rate (FDR; *q* value) was under 0.25, with multiple testing correction applied via the Benjamini-Hochberg (BH) procedure^38^. KEGG is a copyrighted database maintained by Kanehisa Laboratories. In compliance with the journal’s Open Access (CC BY) publishing requirements, we have submitted a formal permission request via the KEGG copyright form (https://www.kegg.jp/feedback/copyright.html; *Publication Detail: Scientific Reports*). KEGG pathway information is cited according to the official KEGG citation policy (https://www.kegg.jp/kegg/kegg1.html), and the permission document will be provided to *Scientific Reports* upon receipt.

### Construction of CRC subtypes

Consensus clustering^17^ is a robust unsupervised method that estimates cluster assignments and evaluates clustering stability through repeated random subsampling of the dataset and aggregation of results across multiple iterations. In this study, consensus clustering was performed with the R package ConsensusClusterPlus^43^ (v1.66.0) using the expression data of the selected key genes to delineate reproducible molecular subtypes in CRC tumors from the TCGA-GTEx-COADREAD dataset. The optimal cluster range (k) was investigated from 2 to 10 across 50 resampling runs, with each run drawing 80% of the samples (clusterAlg = “km”, distance = “euclidean”). Subsequently, we visualized the subtype-specific expression profiles of the key genes as heatmaps and further compared inter-subtype differences using group-based statistical plots.

### Gene set enrichment analysis (GSEA) of CRC subtypes

GSEA^15^ evaluates whether a predefined gene set shows a nonrandom distribution trend within a gene list ranked by its association with a phenotype, thereby inferring the set’s contribution to that phenotype. First, according to the subtype groupings in the TCGA-GTEx-COADREAD cohort, we performed differential expression analysis across subtypes using limma^37^ (version 3.58.1) and defined cluster-related differentially expressed genes (CRDEGs). Volcano plots were created with ggplot2 (v3.5.2) to highlight genes meeting the differential expression threshold (|log2FC| > 1.5; adjusted *p* < 0.05), and heatmaps of these genes were generated using pheatmap (v1.0.13).

Next, all genes in the TCGA-GTEx-COADREAD cohort were ranked by log2FC, and a preranked gene set enrichment analysis (GSEA) was carried out using the clusterProfiler package^42^ (v4.10.1). The analysis applied a random seed of 2025, 1,000 permutations, and gene set size limits of 10 to 500. Gene sets were obtained from the Molecular Signatures Database (MSigDB)^44^, collection *c2.all.v2024.1.Hs.symbols.gmt*. Enrichment significance was determined at an adjusted *p* < 0.05 and false discovery rate (FDR; *q* value) < 0.25, with multiple testing controlled by the Benjamini-Hochberg (BH) procedure.

### Survival curves and clinical characteristics of CRC subtypes

Overall survival (OS) differences among the molecular subtypes in the TCGA-GTEx-COADREAD cohort were assessed using Kaplan-Meier (KM) survival analysis^45^ implemented in the survival R package^39^ (v3.8.3), and corresponding KM survival curves were plotted. Clinical information was subsequently integrated with the assigned subtype labels, and the distribution of clinical features across subtypes was visualized using the ggplot2 package^46^ (v3.5.2).

### PPI network and hub-gene identification

Protein–protein interaction (PPI) networks illustrate functional relationships among proteins involved in key biological processes, including signal transduction, gene expression regulation, cellular metabolism, and cell cycle control. Systematic analysis of PPI networks facilitates the identification of functionally important genes and molecular mechanisms underlying disease development.

In this study, PPI networks were constructed using the STRING database (version 11.5; https://string-db.org/; accessed on September 11, 2025). The CRDEGs were queried with a high-confidence interaction score threshold of 0.900, and only genes with at least one interaction with other nodes were retained for downstream analysis.

The resulting PPI network was visualized using Cytoscape software (version 3.9.1; https://cytoscape.org/). Hub genes were identified using the cytoHubba plugin (version 0.1) by applying five topological ranking algorithms: Maximal Clique Centrality (MCC), Degree, Maximum Neighborhood Component (MNC), Edge Percolated Component (EPC), and Closeness. For each algorithm, the top ten ranked CRDEGs were selected, and the intersection of gene lists from all five methods was visualized using a Venn diagram to define the final set of hub genes.

### Survival analysis of hub genes

To compare OS between high- and low-expression groups of the hub genes in CRC tumors from the TCGA-GTEx-COADREAD cohort, KM survival analysis^45^ was carried out using the R package survival^39^ (v3.8.3), and KM survival curves were plotted. Likewise, OS differences between high- and low-expression groups of the hub genes in the GSE39582 cohort were evaluated using the same KM survival approach implemented with the survival package (v3.8.3). High/low groups were defined by a minimum p-value–based dichotomization using the log-rank test; tests were two-sided and *p* < 0.05 was considered significant.

### Immune infiltration analysis of CRC subtypes (CIBERSORT)

CIBERSORT^16^ is a computational deconvolution method that employs ν-support vector regression to estimate the relative proportions and abundances of immune cell populations from bulk transcriptomic data. Using CIBERSORT together with the leukocyte signature matrix LM22, we estimated immune cell fractions for the CRC tumors in the TCGA-GTEx-COADREAD cohort, retained cell types with nonzero estimated proportions, and visualized the results as stacked bar plots. Next, subtype-wise differences in LM22 immune cell fractions were assessed and displayed using group comparison plots generated with ggplot2 (version 3.5.2).

Immune cell types showing significant inter-subtype differences were retained for further analysis. Spearman correlation coefficients among immune cell populations within each subtype were then calculated, and the resulting relationships were visualized as correlation heatmaps using the pheatmap package (v1.0.13). In addition, associations between hub gene expression and immune cell fractions were evaluated with Spearman correlation analysis and presented as bubble plots created with the ggplot2 package (v3.5.2). Extended visualizations are provided in the Supplementary Information. Unless otherwise specified, tests were two-sided and *p* < 0.05 was considered significant.

### Analyses of immune checkpoint genes (ICGs), ICD, human leukocyte antigen (HLA), TIDE, and IPS

ICGs denote pairs of ligands and receptors that either inhibit or activate immune responses. These genes play essential roles in maintaining immune homeostasis and protecting against autoimmune responses. Immune checkpoint blockade has yielded substantial clinical benefit in several solid malignancies. Relevant references were retrieved from the published literature through searches conducted in PubMed^21^, We curated a set of 47 ICGs (listed in Supplementary Data S5). Differences in their expression between molecular subtypes of CRC tumors in the TCGA-GTEx-COADREAD cohort were evaluated using the Mann-Whitney U test (Wilcoxon rank-sum test). Results were then visualized through group comparison plots.

ICD is a specialized form of regulated cell death (RCD) triggered by cellular stress and capable of provoking adaptive immune responses to antigens released from dying cells.Based on literature retrieved from PubMed^22^, we assembled a panel of 32 ICD-related genes (listed in Supplementary Data S6). Differences in their expression among the identified subtypes were evaluated using the Mann-Whitney U test, and the results were visualized accordingly.

HLA genes are key molecules in the immune system. Using “human leukocyte antigen” as the keyword in GeneCards^35^ (https://www.genecards.org/), we retrieved genes whose symbols begin with “HLA” yielding 33 HLA family genes (Supplementary Data S7). Expression differences of these HLA genes across subtypes in the TCGA-GTEx-COADREAD cohort were evaluated using the Mann-Whitney U test and visualized with group comparison plots.

To obtain TIDE scores for the TCGA-GTEx-COADREAD CRC tumors, we submitted the cohort’s expression matrix to the TIDE web portal^8,47^. The TIDE algorithm predicts potential responses to immune checkpoint inhibitors (ICIs) and examines the relationship between gene expression and patient survival across tumors. Using TIDE outputs, we compared TIDE scores between the identified subtypes by applying the Mann-Whitney U test.

Immunogenicity denotes the ability of an antigen or epitope to engage T- and B-cell receptors and thereby initiate humoral and/or cell-mediated immune responses; such antigens are referred to as immunogens. This property is influenced by multiple gene programs, including those governing effector cell activity, major histocompatibility complex (MHC) expression, immunoregulatory molecules, and immunosuppressive cell populations. Using the gene expression matrix of CRC tumors from the TCGA-GTEx-COADREAD cohort, we calculated five immunogenicity-related scores with the R package IOBR (v0.99.0)^48^: the MHC score, effector cells (EC), suppressor cells (SC), immune checkpoints (CP), and the integrated immunophenoscore (IPS). Differences in these scores between molecular subtypes were assessed with the Mann-Whitney U test.

### Statistical analysis

All data preprocessing and statistical analyses were conducted in R (v4.3.3). Unless otherwise stated, continuous variables between two groups were compared using the independent Student’s *t*-test for normally distributed data and the Mann-Whitney U test (Wilcoxon rank-sum test) when normality was not satisfied. Associations among molecular features were examined by Spearman correlation analysis. Unless specified otherwise, all *p-value*s were two-sided, and results with *p* < 0.05 were regarded as statistically significant.

## Supplementary Information

Below is the link to the electronic supplementary material.

Supplementary Material 1

Supplementary Material 2

Supplementary Material 3

Supplementary Material 4

Supplementary Material 5

Supplementary Material 6

Supplementary Material 7

Supplementary Material 8

Supplementary Material 9

Supplementary Material 10

Supplementary Material 11

Supplementary Material 12

Supplementary Material 13

Supplementary Material 14

Supplementary Material 15

Supplementary Material 16

Supplementary Material 17

## Data Availability

All data analyzed in this study are publicly available. TCGA-COAD/READ datasets were obtained from The Cancer Genome Atlas (https://portal.gdc.cancer.gov/). Normal tissue data were retrieved from the GTEx database (https://gtexportal.org/). The validation dataset GSE39582 was downloaded from the Gene Expression Omnibus (https://www.ncbi.nlm.nih.gov/geo/). All R scripts and processed data supporting the findings are available from the corresponding author upon reasonable request.
