# Supplementary figures and images for: Deubiquitination-related genes define immune subtypes of colorectal cancer and are associated with prognosis and immunotherapy-related signatures
Source: Sci Rep. 2026 Jan 8;16:4862. doi: 10.1038/s41598-026-35271-5 (PMC12873191; doi:10.1038/s41598-026-35271-5)

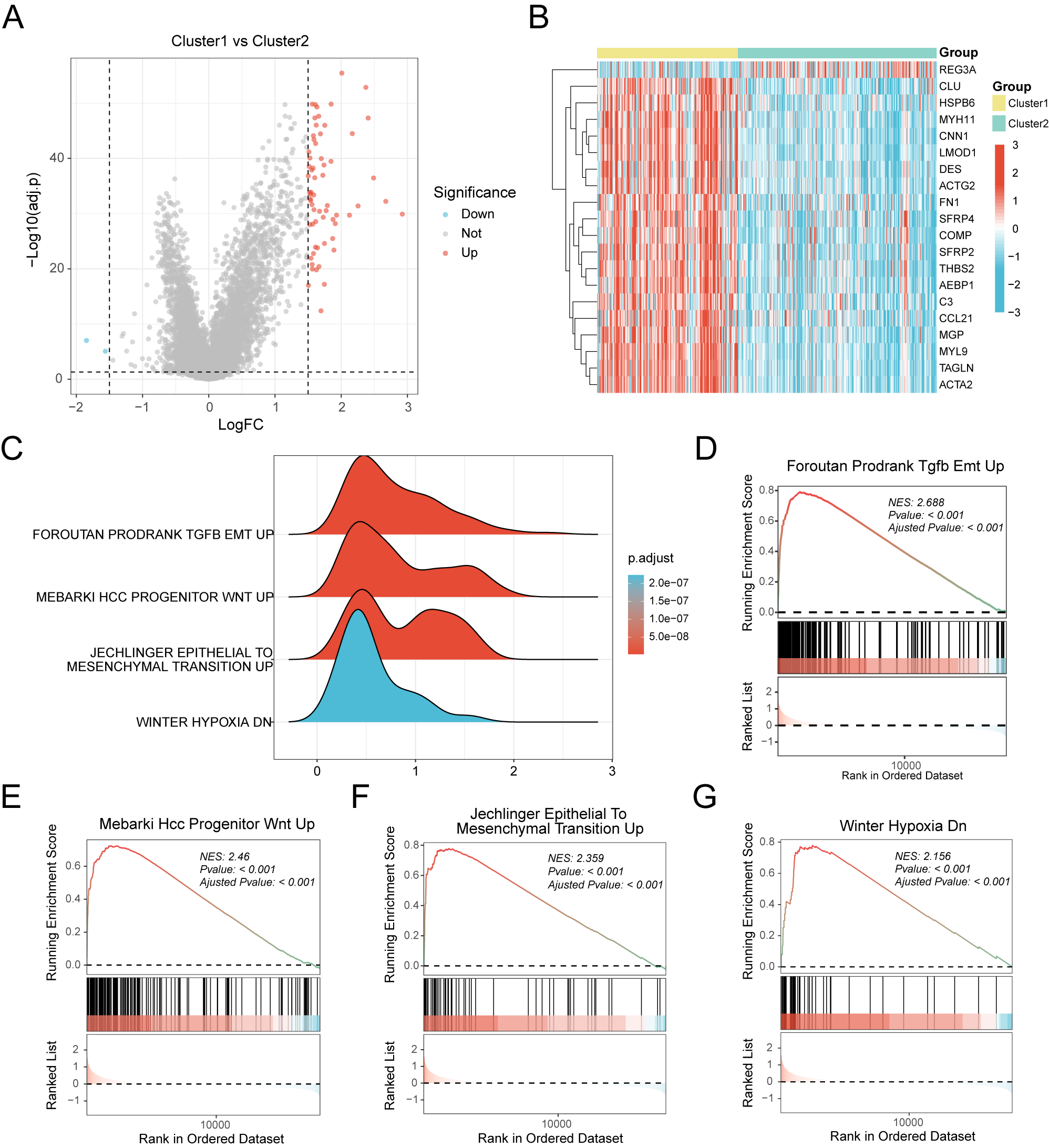

Supplement: Supplementary file 2 — Supplementary Material 2 [file 41598_2026_35271_MOESM2_ESM.tif]

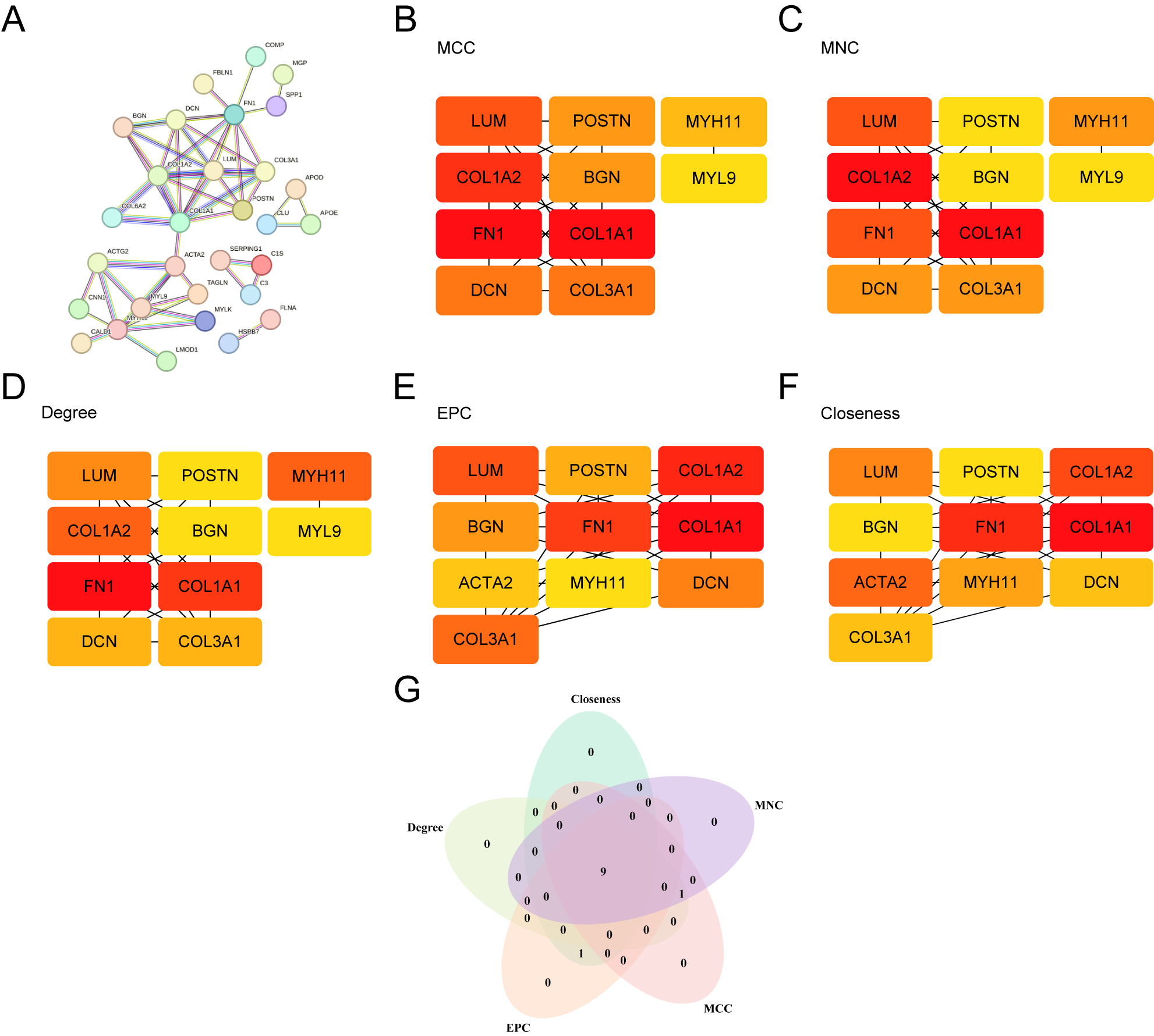

Supplement: Supplementary file 3 — Supplementary Material 3 [file 41598_2026_35271_MOESM3_ESM.tif]

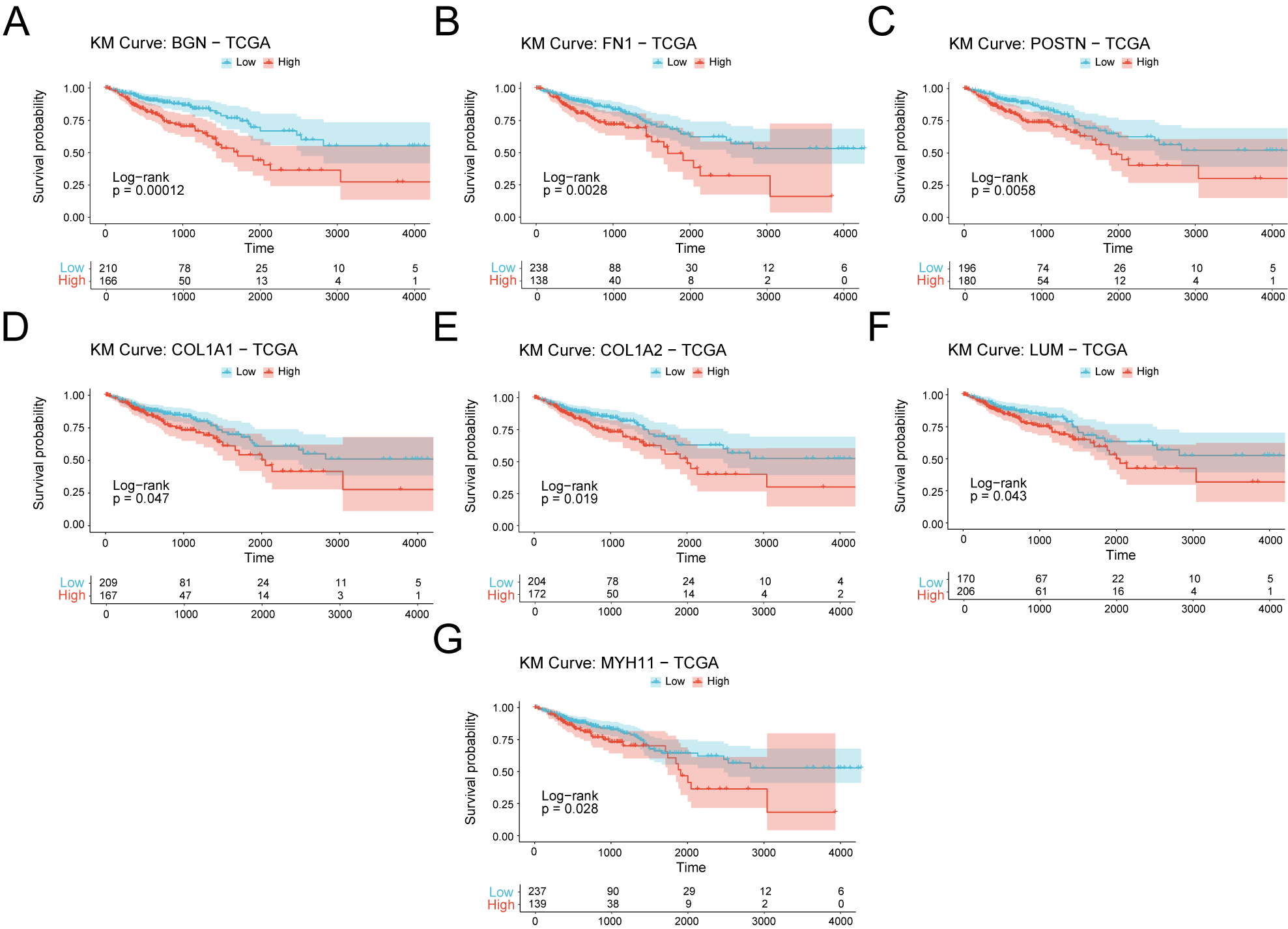

Supplement: Supplementary file 4 — Supplementary Material 4 [file 41598_2026_35271_MOESM4_ESM.tif]

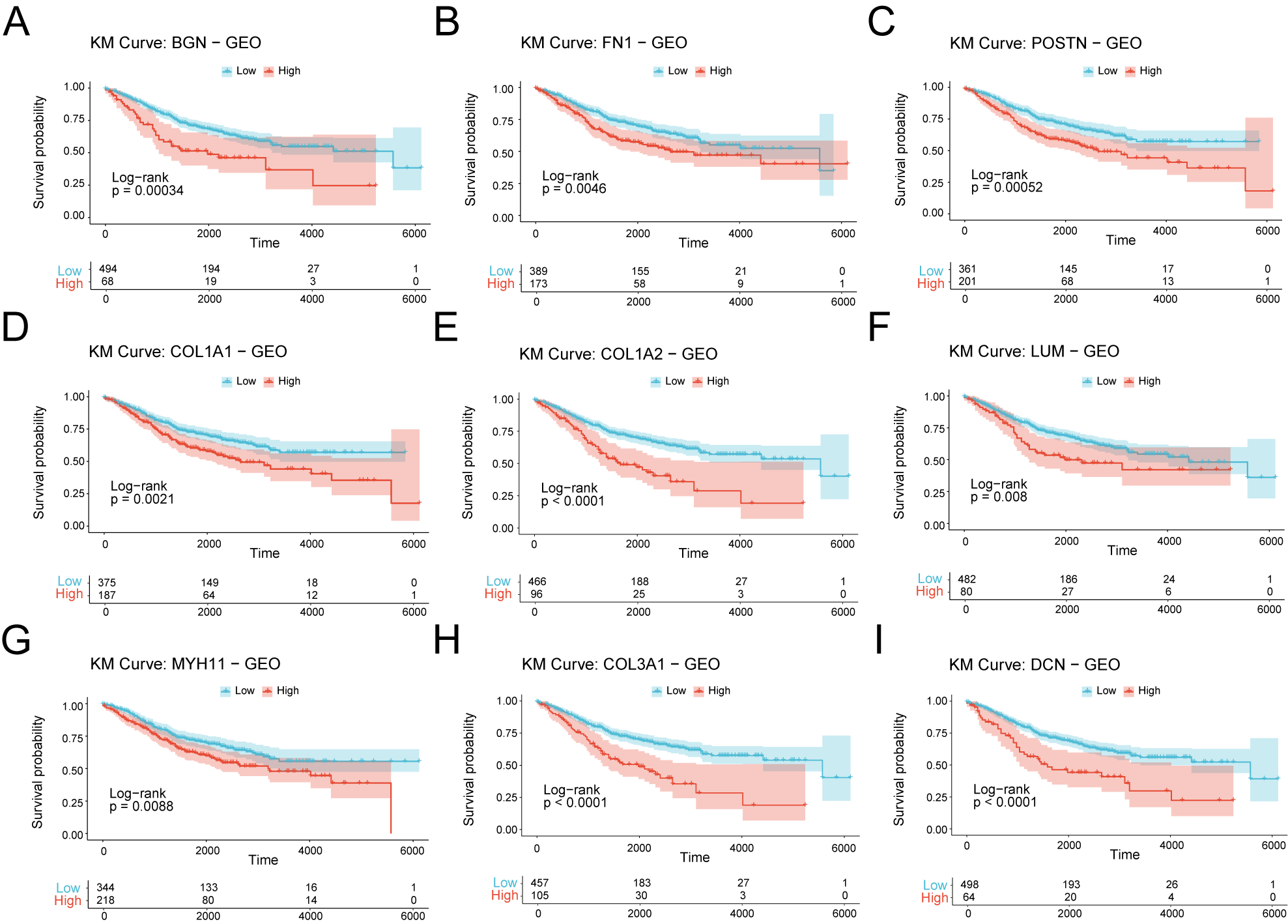

Supplement: Supplementary file 5 — Supplementary Material 5 [file 41598_2026_35271_MOESM5_ESM.tif]

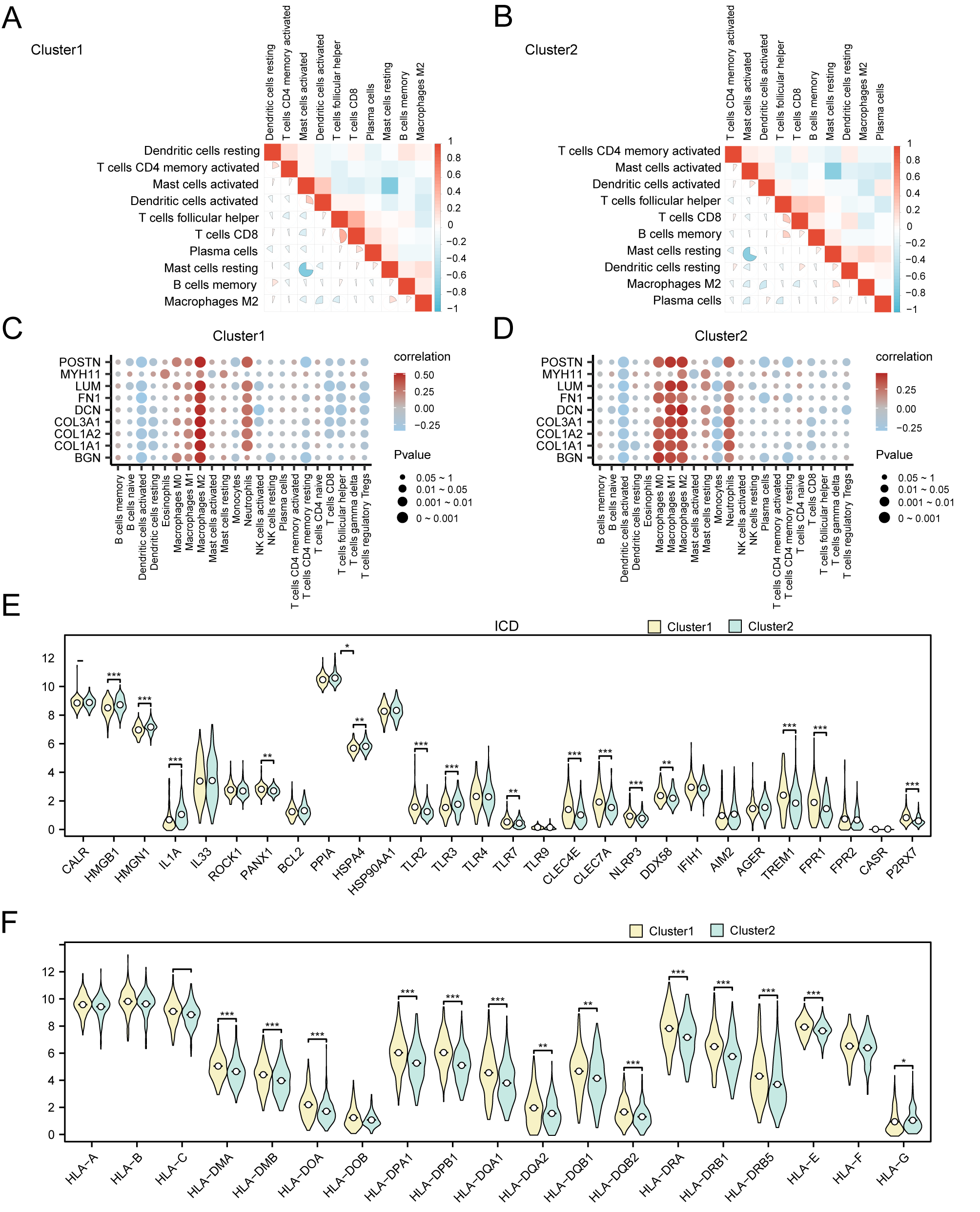

Supplement: Supplementary file 6 — Supplementary Material 6 [file 41598_2026_35271_MOESM6_ESM.tif]
