# Supplementary material for: Deubiquitination-related genes define immune subtypes of colorectal cancer and are associated with prognosis and immunotherapy-related signatures
Source: Sci Rep. 2026 Jan 8;16:4862. doi: 10.1038/s41598-026-35271-5 (PMC12873191; doi:10.1038/s41598-026-35271-5)
Supplement: Supplementary file 7 — Supplementary Material 7 [file 41598_2026_35271_MOESM7_ESM.docx]

**Table 2** **GEO Microarray Chip Information**

|  | GSE39582 |
| --- | --- |
| Platform | GPL570 |
| Species | Homo sapiens |
| Tissue | Colorectal Cancer tumor |
| Samples in CRC group | 566 |
| Samples in Normal group | 19 |
| Reference | PMID：3700391 |

GEO，Gene Expression Omnibus；CRC，Colorectal Cancer.
