# Supplementary material for: Deubiquitination-related genes define immune subtypes of colorectal cancer and are associated with prognosis and immunotherapy-related signatures
Source: Sci Rep. 2026 Jan 8;16:4862. doi: 10.1038/s41598-026-35271-5 (PMC12873191; doi:10.1038/s41598-026-35271-5)
Supplement: Supplementary file 8 — Supplementary Material 8 [file 41598_2026_35271_MOESM8_ESM.docx]

**Table 3 Results of GO and KEGG Enrichment Analysis for Key Genes**

| Ontology | ID | Description | GeneRatio | BgRatio | pvalue | p.adjust | qvalue |
| --- | --- | --- | --- | --- | --- | --- | --- |
| BP | GO:0044839 | cell cycle G2/M phase transition | 6/17 | 155/18870 | 3.20E-09 | 2.83E-06 | 1.55E-06 |
| BP | GO:1902749 | regulation of cell cycle G2/M phase transition | 5/17 | 116/18870 | 4.70E-08 | 2.07E-05 | 1.14E-05 |
| BP | GO:0000086 | G2/M transition of mitotic cell cycle | 5/17 | 140/18870 | 1.21E-07 | 2.86E-05 | 1.57E-05 |
| BP | GO:0007093 | mitotic cell cycle checkpoint signaling | 5/17 | 142/18870 | 1.29E-07 | 2.86E-05 | 1.57E-05 |
| BP | GO:0010948 | negative regulation of cell cycle process | 6/17 | 320/18870 | 2.40E-07 | 4.24E-05 | 2.33E-05 |
| CC | GO:0031616 | spindle pole centrosome | 2/17 | 14/19886 | 6.22E-05 | 6.52E-03 | 3.67E-03 |
| CC | GO:1990391 | DNA repair complex | 2/17 | 22/19886 | 1.57E-04 | 6.52E-03 | 3.67E-03 |
| CC | GO:1902554 | serine/threonine protein kinase complex | 3/17 | 128/19886 | 1.66E-04 | 6.52E-03 | 3.67E-03 |
| CC | GO:1902911 | protein kinase complex | 3/17 | 148/19886 | 2.54E-04 | 7.51E-03 | 4.22E-03 |
| CC | GO:0000922 | spindle pole | 3/17 | 176/19886 | 4.23E-04 | 9.98E-03 | 5.61E-03 |
| MF | GO:0044389 | ubiquitin-like protein ligase binding | 4/17 | 327/18496 | 1.90E-04 | 1.96E-02 | 9.61E-03 |
| MF | GO:0016538 | cyclin-dependent protein serine/threonine kinase regulator activity | 2/17 | 50/18496 | 9.49E-04 | 3.70E-02 | 1.82E-02 |
| MF | GO:0019887 | protein kinase regulator activity | 3/17 | 226/18496 | 1.08E-03 | 3.70E-02 | 1.82E-02 |
| MF | GO:0019207 | kinase regulator activity | 3/17 | 256/18496 | 1.54E-03 | 3.97E-02 | 1.95E-02 |
| KEGG | hsa04110 | Cell cycle | 4/13 | 158/9392 | 4.90E-05 | 2.01E-03 | 1.50E-03 |
| KEGG | hsa04218 | Cellular senescence | 3/13 | 157/9392 | 1.16E-03 | 2.38E-02 | 1.77E-02 |
| KEGG | hsa05166 | Human T-cell leukemia virus 1 infection | 3/13 | 224/9392 | 3.21E-03 | 4.38E-02 | 3.26E-02 |
| KEGG | hsa04115 | p53 signaling pathway | 2/13 | 75/9392 | 4.64E-03 | 4.50E-02 | 3.35E-02 |
| KEGG | hsa05012 | Parkinson disease | 3/13 | 271/9392 | 5.48E-03 | 4.50E-02 | 3.35E-02 |

GO，Gene Ontology；BP，Biological Process；CC，Cellular Component；MF，Molecular Function；KEGG，Kyoto Encyclopedia of Genes and Genomes.
