# Supplementary material for: Deubiquitination-related genes define immune subtypes of colorectal cancer and are associated with prognosis and immunotherapy-related signatures
Source: Sci Rep. 2026 Jan 8;16:4862. doi: 10.1038/s41598-026-35271-5 (PMC12873191; doi:10.1038/s41598-026-35271-5)
Supplement: Supplementary file 9 — Supplementary Material 9 [file 41598_2026_35271_MOESM9_ESM.docx]

**Table 4 Results of GSEA for Cluster**

| ID | setSize | enrichmentScore | NES | p.adjust | qvalue |
| --- | --- | --- | --- | --- | --- |
| CARRILLOREIXACH_MRS3_VS_LOWER_RISK_HEPATOBLASTOMA_DN | 153 | 0.819330 | 2.725669 | 1.00E-10 | 1.82E-09 |
| FOROUTAN_INTEGRATED_TGFB_EMT_UP | 118 | 0.838852 | 2.722116 | 1.00E-10 | 1.82E-09 |
| FOROUTAN_TGFB_EMT_UP | 189 | 0.798700 | 2.721251 | 1.00E-10 | 1.82E-09 |
| FOROUTAN_PRODRANK_TGFB_EMT_UP | 182 | 0.791337 | 2.688085 | 1.00E-10 | 1.82E-09 |
| TURASHVILI_BREAST_LOBULAR_CARCINOMA_VS_DUCTAL_NORMAL_UP | 66 | 0.877605 | 2.646231 | 1.00E-10 | 1.82E-09 |
| TURASHVILI_BREAST_LOBULAR_CARCINOMA_VS_LOBULAR_NORMAL_DN | 71 | 0.863166 | 2.644957 | 1.00E-10 | 1.82E-09 |
| HOLLERN_EMT_BREAST_TUMOR_UP | 137 | 0.801390 | 2.626576 | 1.00E-10 | 1.82E-09 |
| HELLEBREKERS_SILENCED_DURING_TUMOR_ANGIOGENESIS | 76 | 0.798020 | 2.460953 | 1.00E-10 | 1.82E-09 |
| MEBARKI_HCC_PROGENITOR_WNT_UP | 178 | 0.724547 | 2.459963 | 1.00E-10 | 1.82E-09 |
| WILCOX_RESPONSE_TO_PROGESTERONE_DN | 59 | 0.838575 | 2.451215 | 1.00E-10 | 1.82E-09 |
| REACTOME_ASSEMBLY_OF_COLLAGEN_FIBRILS_AND_OTHER_MULTIMERIC_STRUCTURES | 61 | 0.825342 | 2.427540 | 1.00E-10 | 1.82E-09 |
| MANALO_HYPOXIA_UP | 197 | 0.705193 | 2.421365 | 1.00E-10 | 1.82E-09 |
| MCBRYAN_PUBERTAL_TGFB1_TARGETS_UP | 165 | 0.715567 | 2.408773 | 1.00E-10 | 1.82E-09 |
| FRIDMAN_SENESCENCE_UP | 75 | 0.769541 | 2.372654 | 1.00E-10 | 1.82E-09 |
| JECHLINGER_EPITHELIAL_TO_MESENCHYMAL_TRANSITION_UP | 67 | 0.779296 | 2.359124 | 1.00E-10 | 1.82E-09 |
| MEBARKI_HCC_PROGENITOR_WNT_UP_BLOCKED_BY_FZD8CRD | 113 | 0.716657 | 2.321164 | 1.00E-10 | 1.82E-09 |
| THUM_SYSTOLIC_HEART_FAILURE_UP | 392 | 0.645400 | 2.319399 | 1.00E-10 | 1.82E-09 |
| CHEN_LVAD_SUPPORT_OF_FAILING_HEART_UP | 96 | 0.730825 | 2.315225 | 1.00E-10 | 1.82E-09 |
| VERRECCHIA_EARLY_RESPONSE_TO_TGFB1 | 54 | 0.795822 | 2.290420 | 1.00E-10 | 1.82E-09 |
| WINTER_HYPOXIA_DN | 45 | 0.779113 | 2.156261 | 1.73E-08 | 2.24E-07 |

GSEA，Gene Set Enrichment Analysis.
