# Supplementary material for: Deubiquitination-related genes define immune subtypes of colorectal cancer and are associated with prognosis and immunotherapy-related signatures
Source: Sci Rep. 2026 Jan 8;16:4862. doi: 10.1038/s41598-026-35271-5 (PMC12873191; doi:10.1038/s41598-026-35271-5)
Supplement: Supplementary file 17 — Supplementary Material 17 [file 41598_2026_35271_MOESM17_ESM.docx]

# Deubiquitination-Related Genes Define Immune Subtypes of Colorectal Cancer and Predict Prognosis and Immunotherapy Response

Yiwei Xu¹,† Zhiyong Mo¹,† Qing Jiang¹, Jingjing Pan¹, Qi Xu², and Juwen Jia¹,*¹ Department of General Surgery, Second People’s Hospital of Longgang District, Shenzhen, Guangdong, China
² Department of Oncology, West Coast Second Hospital, Qingdao University Medical Group, Qingdao, Shandong Province, China

* Corresponding author: Mr Juwen Jia MS, Associate Chief Physician, Department of General Surgery, Second People’s Hospital of Longgang District, Shenzhen, No. 175 Jihua Road, Buji Street, Longgang District, Shenzhen 518112, Guangdong Province, China. Email: xxyx4586@163.com

† These authors contributed equally to this work.

This file contains Supplementary Figures S1–S5 and Supplementary Tables S1–S3.
Supplementary Data S1–S7 are provided as separate CSV files and are not included within this document.

# Abbreviations

GO, Gene Ontology;
KEGG, Kyoto Encyclopedia of Genes and Genomes;
BP, biological process;
CC, cellular component;
MF, molecular function;
TCGA, The Cancer Genome Atlas;

GEO, Gene Expression Omnibus;
GTEx, Genotype-Tissue Expression;
COADREAD, Colon and Rectal Adenocarcinoma;
CRC, colorectal cancer;

DEGs, differentially expressed genes;
CRDEGs, cluster-related differentially expressed genes;
PPI, protein-protein interaction;
STRING, Search Tool for the Retrieval of Interacting Genes/Proteins;
MCC, Maximal Clique Centrality;
MNC, Maximum Neighborhood Component;
EPC, Edge Percolated Component;
OS, overall survival;
KM, Kaplan-Meier;
ICD, immunogenic cell death;
HLA, human leukocyte antigen;
IPS, Immunophenoscore;
TIDE, Tumor Immune Dysfunction and Exclusion;

# Supplementary Figure S1


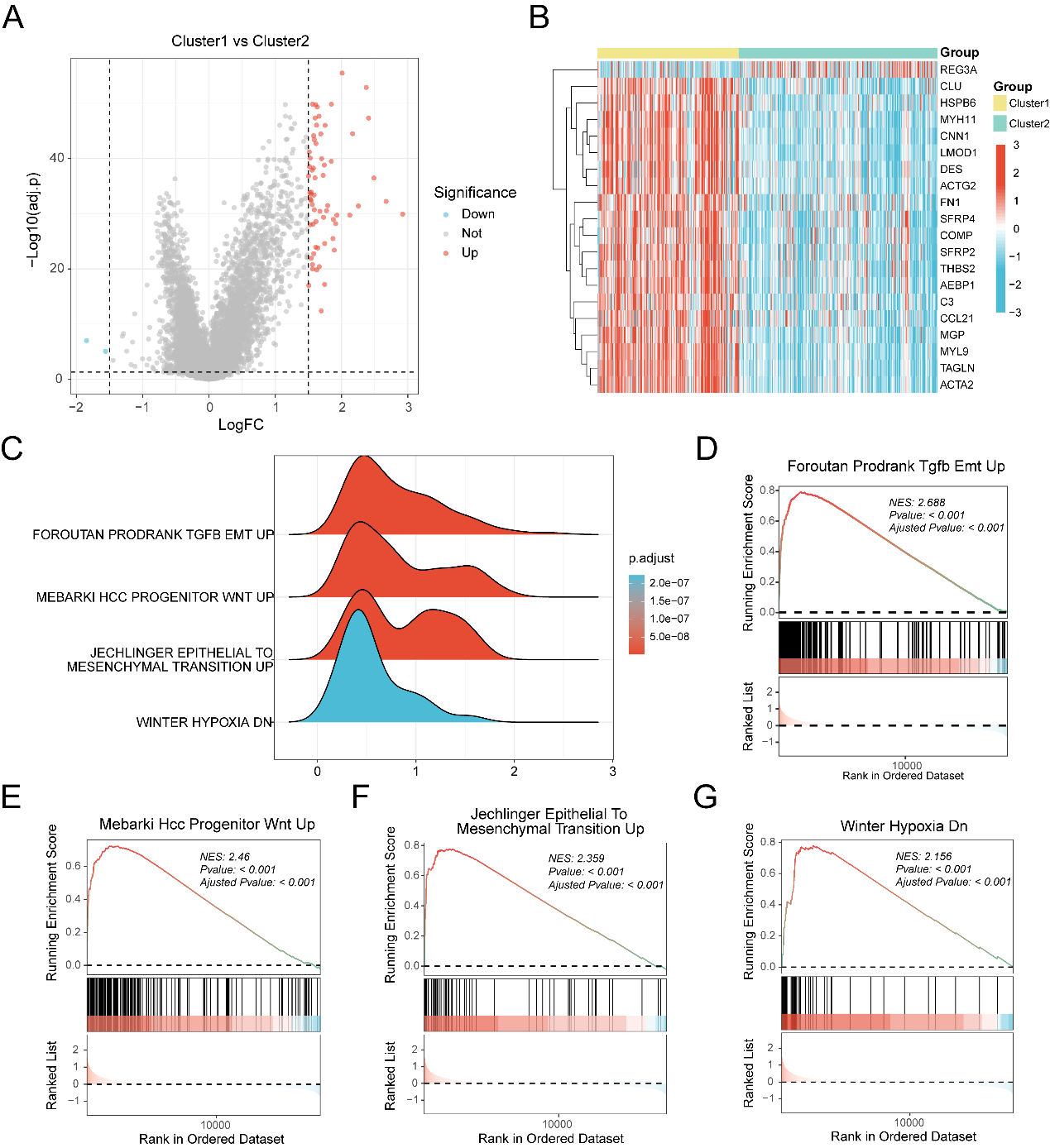


Supplementary Figure S1. Differential expression and GSEA results between CRC subtypes.

(A) Volcano plot of DEGs between subtype A (Cluster1) and subtype B (Cluster2) in the TCGA-GTEx-COADREAD dataset. (B) Heatmap showing the expression patterns of CRDEGs (red = high, blue = low). (C) Ridge plots for four biological functions. (D-G) GSEA indicates significant enrichment of gene sets: Foroutan Prodrank Tgfb Emt Up (D), Mebarki Hcc Progenitor Wnt Up (E), Jechlinger Epithelial to Mesenchymal Transition Up (F), and Winter Hypoxia Dn (G).

# Supplementary Figure S2


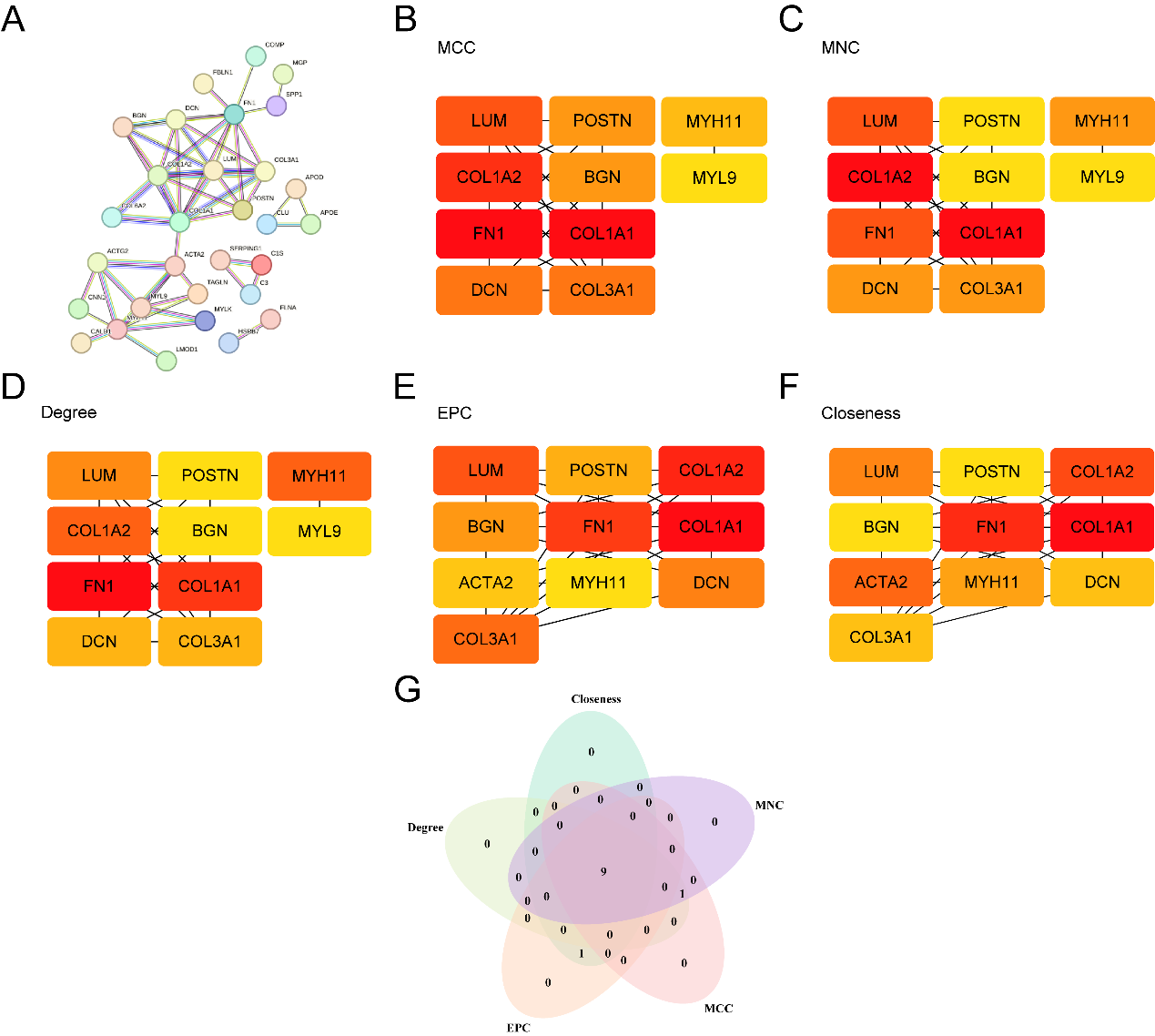


Supplementary Figure S2. PPI network construction and hub gene prioritization.

(A) STRING-derived PPI network of CRDEGs. (B-F) PPI subnetworks formed by the top 10 CRDEGs ranked by each cytoHubba algorithm: MCC (B), MNC (C), Degree (D), EPC (E), and Closeness (F). (G) Venn diagram of the top-10 gene sets across the five cytoHubba algorithms, used to define candidate hub genes. The PPI network was constructed using STRING (https://string-db.org/) and visualized in Cytoscape (version 3.9.1; https://cytoscape.org/) with the cytoHubba plugin (version 0.1).

# Supplementary Figure S3


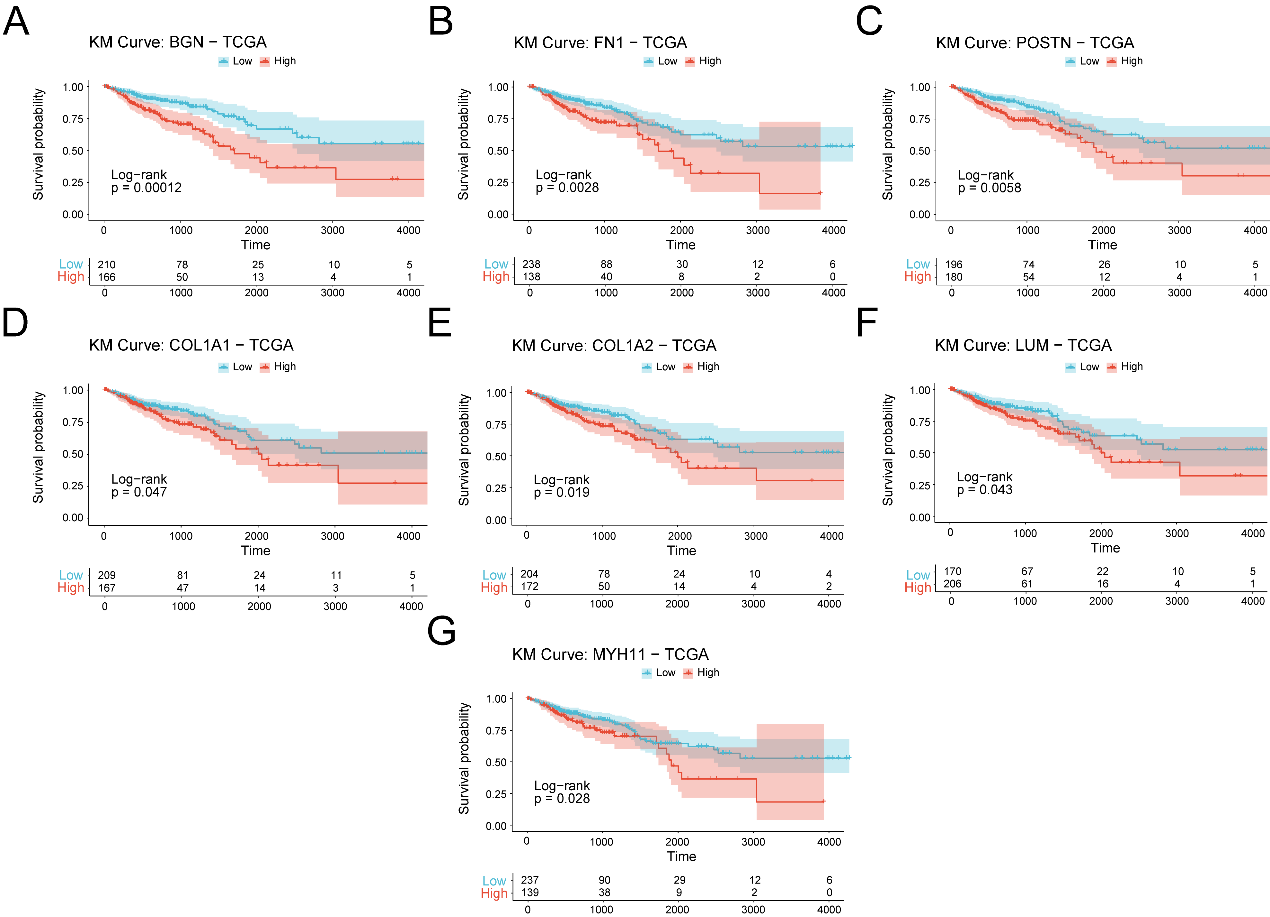


Supplementary Figure S3. Survival analysis of hub genes in the TCGA cohort.

(A-G) KM survival curves for OS comparing high- versus low-expression groups of the hub genes BGN (A), FN1 (B), POSTN (C), COL1A1 (D), COL1A2 (E), LUM (F), and MYH11 (G) in CRC samples from the TCGA-GTEx-COADREAD dataset. Red indicates the high-expression group and blue indicates the low-expression group.

# Supplementary Figure S4


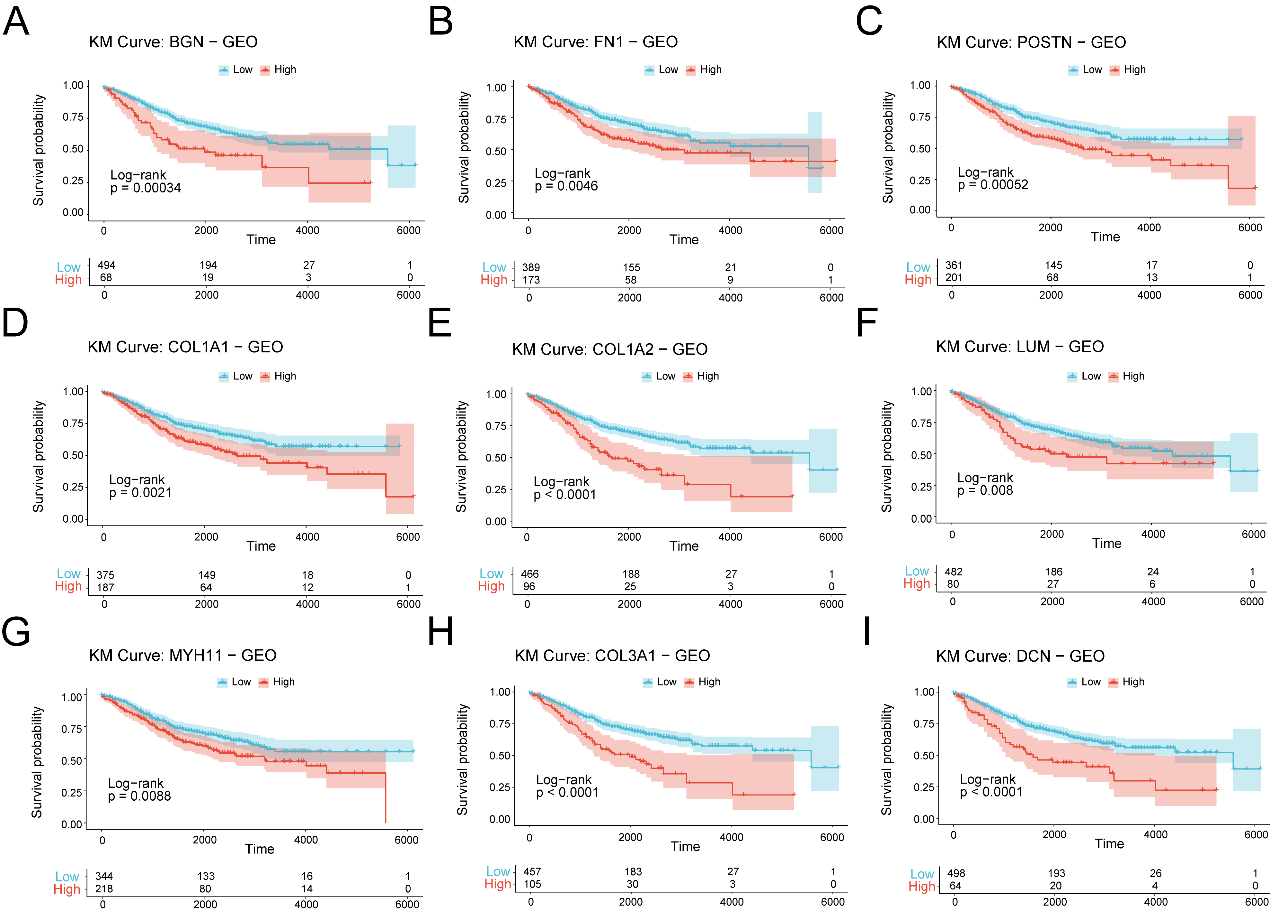


Supplementary Figure S4. Survival analysis of hub genes in the GSE39582 cohort.

(A-I) KM survival curves for OS comparing high- versus low-expression groups of the hub genes BGN (A), FN1 (B), POSTN (C), COL1A1 (D), COL1A2 (E), LUM (F), MYH11 (G), COL3A1 (H), and DCN (I) in CRC samples from the GSE39582 dataset. Red indicates the high-expression group and blue indicates the low-expression group.

# Supplementary Figure S5


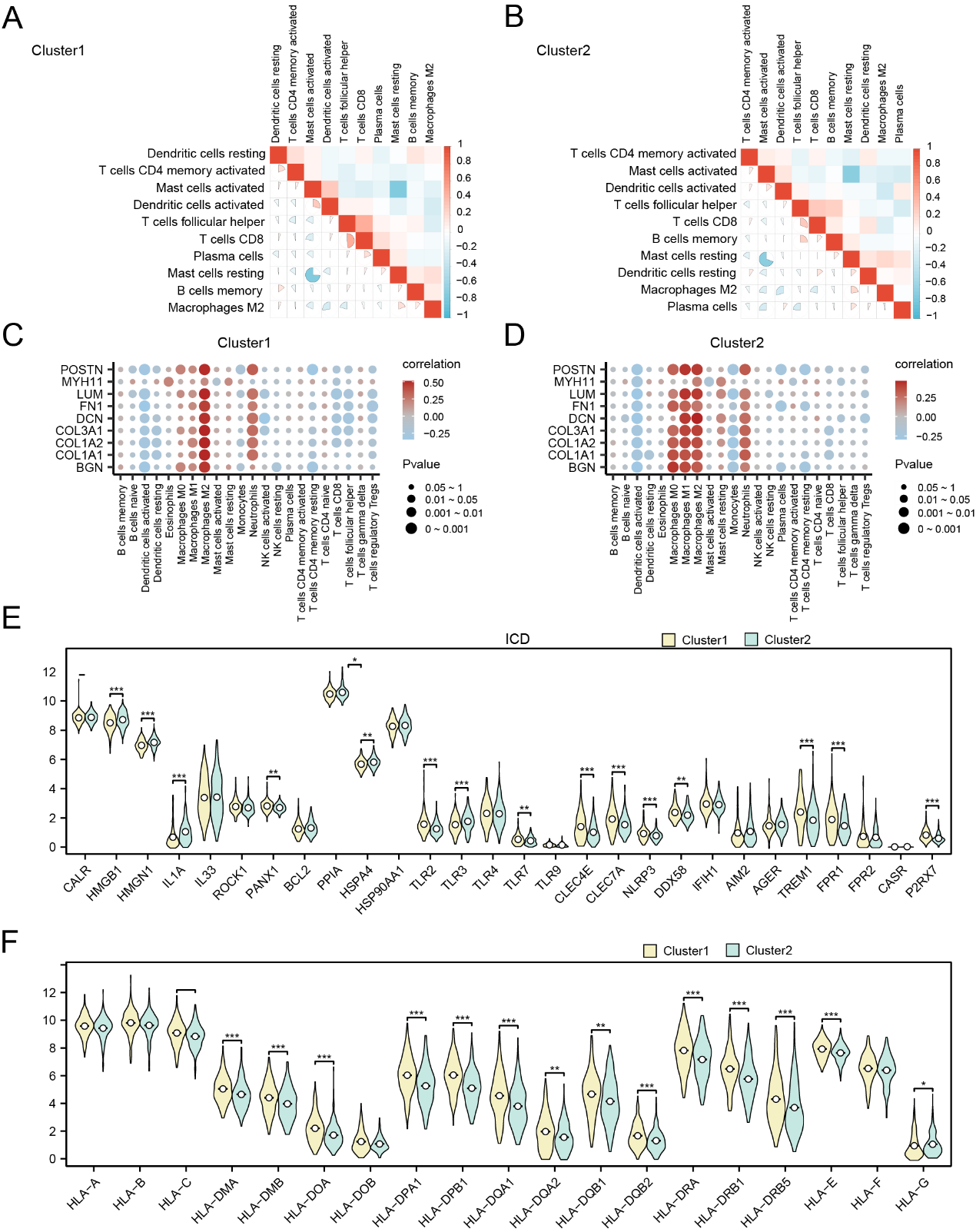


Supplementary Figure S5. Extended immune analyses for CRC molecular subtypes.
(A–B) Within-subtype correlation heatmaps for significantly different LM22 immune-cell populations in Cluster 1 (A) and Cluster 2 (B). Red indicates positive and blue negative Spearman correlations; color intensity reflects coefficient magnitude. (C–D) Associations between hub-gene expression and immune-cell fractions within each subtype, shown as bubble plots for Cluster 1 (C) and Cluster 2 (D). Bubble size encodes −log10(*p*), and color encodes the correlation coefficient (red = positive, blue = negative). (E) Differential expression of immunogenic cell death (ICD) genes between subtypes, displayed as violin plots. (F) Differential expression of HLA family genes between subtypes, displayed as violin plots. Between-subtype comparisons were assessed with the Wilcoxon rank-sum test; correlations used two-sided Spearman tests. Significance annotations: ns, *p* ≥ 0.05; *, *p* < 0.05; **, *p* < 0.01; ***, *p* < 0.001. Color scheme: yellow, Cluster 1; green, Cluster 2.

# Supplementary Table S1. GEO Microarray Chip Information

|  | GSE39582 |
| --- | --- |
| Platform | GPL570 |
| Species | Homo sapiens |
| Tissue | Colorectal Cancer tumor |
| Samples in CRC group | 566 |
| Samples in Control group | 19 |
| Reference | PMID: 23700391 |

# Supplementary Table S2. Results of GO and KEGG Enrichment Analysis for key genes

| Ontology | ID | Description | GeneRatio | BgRatio | pvalue | p.adjust | qvalue |
| --- | --- | --- | --- | --- | --- | --- | --- |
| BP | GO:0044839 | cell cycle G2/M phase transition | 6/17 | 155/18870 | 3.20E-09 | 2.83E-06 | 1.55E-06 |
| BP | GO:1902749 | regulation of cell cycle G2/M phase transition | 5/17 | 116/18870 | 4.70E-08 | 2.07E-05 | 1.14E-05 |
| BP | GO:0000086 | G2/M transition of mitotic cell cycle | 5/17 | 140/18870 | 1.21E-07 | 2.86E-05 | 1.57E-05 |
| BP | GO:0007093 | mitotic cell cycle checkpoint signaling | 5/17 | 142/18870 | 1.29E-07 | 2.86E-05 | 1.57E-05 |
| BP | GO:0010948 | negative regulation of cell cycle process | 6/17 | 320/18870 | 2.40E-07 | 4.24E-05 | 2.33E-05 |
| CC | GO:0031616 | spindle pole centrosome | 2/17 | 14/19886 | 6.22E-05 | 6.52E-03 | 3.67E-03 |
| CC | GO:1990391 | DNA repair complex | 2/17 | 22/19886 | 1.57E-04 | 6.52E-03 | 3.67E-03 |
| CC | GO:1902554 | serine/threonine protein kinase complex | 3/17 | 128/19886 | 1.66E-04 | 6.52E-03 | 3.67E-03 |
| CC | GO:1902911 | protein kinase complex | 3/17 | 148/19886 | 2.54E-04 | 7.51E-03 | 4.22E-03 |
| CC | GO:0000922 | spindle pole | 3/17 | 176/19886 | 4.23E-04 | 9.98E-03 | 5.61E-03 |
| MF | GO:0044389 | ubiquitin-like protein ligase binding | 4/17 | 327/18496 | 1.90E-04 | 1.96E-02 | 9.61E-03 |
| MF | GO:0016538 | cyclin-dependent protein serine/threonine kinase regulator activity | 2/17 | 50/18496 | 9.49E-04 | 3.70E-02 | 1.82E-02 |
| MF | GO:0019887 | protein kinase regulator activity | 3/17 | 226/18496 | 1.08E-03 | 3.70E-02 | 1.82E-02 |
| MF | GO:0019207 | kinase regulator activity | 3/17 | 256/18496 | 1.54E-03 | 3.97E-02 | 1.95E-02 |
| KEGG | hsa04110 | Cell cycle | 4/13 | 158/9392 | 4.90E-05 | 2.01E-03 | 1.50E-03 |
| KEGG | hsa04218 | Cellular senescence | 3/13 | 157/9392 | 1.16E-03 | 2.38E-02 | 1.77E-02 |
| KEGG | hsa05166 | Human T-cell leukemia virus 1 infection | 3/13 | 224/9392 | 3.21E-03 | 4.38E-02 | 3.26E-02 |
| KEGG | hsa04115 | p53 signaling pathway | 2/13 | 75/9392 | 4.64E-03 | 4.50E-02 | 3.35E-02 |
| KEGG | hsa05012 | Parkinson disease | 3/13 | 271/9392 | 5.48E-03 | 4.50E-02 | 3.35E-02 |

# Supplementary Table S3. Results of GSEA for Cluster

| ID | setSize | enrichmentScore | NES | p.adjust | qvalue |
| --- | --- | --- | --- | --- | --- |
| CARRILLOREIXACH_MRS3_VS_LOWER_RISK_HEPATOBLASTOMA_DN | 153 | 0.819330 | 2.725669 | 1.00E-10 | 1.82E-09 |
| FOROUTAN_INTEGRATED_TGFB_EMT_UP | 118 | 0.838852 | 2.722116 | 1.00E-10 | 1.82E-09 |
| FOROUTAN_TGFB_EMT_UP | 189 | 0.798700 | 2.721251 | 1.00E-10 | 1.82E-09 |
| FOROUTAN_PRODRANK_TGFB_EMT_UP | 182 | 0.791337 | 2.688085 | 1.00E-10 | 1.82E-09 |
| TURASHVILI_BREAST_LOBULAR_CARCINOMA_VS_DUCTAL_NORMAL_UP | 66 | 0.877605 | 2.646231 | 1.00E-10 | 1.82E-09 |
| TURASHVILI_BREAST_LOBULAR_CARCINOMA_VS_LOBULAR_NORMAL_DN | 71 | 0.863166 | 2.644957 | 1.00E-10 | 1.82E-09 |
| HOLLERN_EMT_BREAST_TUMOR_UP | 137 | 0.801390 | 2.626576 | 1.00E-10 | 1.82E-09 |
| HELLEBREKERS_SILENCED_DURING_TUMOR_ANGIOGENESIS | 76 | 0.798020 | 2.460953 | 1.00E-10 | 1.82E-09 |
| MEBARKI_HCC_PROGENITOR_WNT_UP | 178 | 0.724547 | 2.459963 | 1.00E-10 | 1.82E-09 |
| WILCOX_RESPONSE_TO_PROGESTERONE_DN | 59 | 0.838575 | 2.451215 | 1.00E-10 | 1.82E-09 |
| REACTOME_ASSEMBLY_OF_COLLAGEN_FIBRILS_AND_OTHER_MULTIMERIC_STRUCTURES | 61 | 0.825342 | 2.427540 | 1.00E-10 | 1.82E-09 |
| MANALO_HYPOXIA_UP | 197 | 0.705193 | 2.421365 | 1.00E-10 | 1.82E-09 |
| MCBRYAN_PUBERTAL_TGFB1_TARGETS_UP | 165 | 0.715567 | 2.408773 | 1.00E-10 | 1.82E-09 |
| FRIDMAN_SENESCENCE_UP | 75 | 0.769541 | 2.372654 | 1.00E-10 | 1.82E-09 |
| JECHLINGER_EPITHELIAL_TO_MESENCHYMAL_TRANSITION_UP | 67 | 0.779296 | 2.359124 | 1.00E-10 | 1.82E-09 |
| MEBARKI_HCC_PROGENITOR_WNT_UP_BLOCKED_BY_FZD8CRD | 113 | 0.716657 | 2.321164 | 1.00E-10 | 1.82E-09 |
| THUM_SYSTOLIC_HEART_FAILURE_UP | 392 | 0.645400 | 2.319399 | 1.00E-10 | 1.82E-09 |
| CHEN_LVAD_SUPPORT_OF_FAILING_HEART_UP | 96 | 0.730825 | 2.315225 | 1.00E-10 | 1.82E-09 |
| VERRECCHIA_EARLY_RESPONSE_TO_TGFB1 | 54 | 0.795822 | 2.290420 | 1.00E-10 | 1.82E-09 |
| WINTER_HYPOXIA_DN | 45 | 0.779113 | 2.156261 | 1.73E-08 | 2.24E-07 |
